# Gene expression, proteome and calcium signaling alterations in immortalized hippocampal astrocytes from an Alzheimer’s disease mouse model

**DOI:** 10.1038/s41419-018-1264-8

**Published:** 2019-01-10

**Authors:** Francesca Rocchio, Laura Tapella, Marcello Manfredi, Mariangela Chisari, Francesca Ronco, Federico Alessandro Ruffinatti, Eleonora Conte, Pier Luigi Canonico, Maria Angela Sortino, Mariagrazia Grilli, Emilio Marengo, Armando A. Genazzani, Dmitry Lim

**Affiliations:** 1 Department of Pharmaceutical Sciences, Università degli Studi del Piemonte Orientale, Novara, Italy; 2 Department of Sciences and Technological Innovation, Università degli Studi del Piemonte Orientale, Alessandria, Italy; 3 ISALIT S.r.l., Spin-off of Università degli Studi del Piemonte Orientale, Novara, Italy; 4 Department of Biomedical and Biotechnological Sciences, Section of Pharmacology, University of Catania, Via Santa Sofia, 97, 95123 Catania, Italy; 5 Present Address: International Center for T1D, Pediatric Clinic Research Center Fondazione Romeo ed Enrica Invernizzi, Department of Biomedical and Clinical Science L. Sacco, University of Milan, Milan, Italy

**Keywords:** Astrocyte, Alzheimer's disease

## Abstract

Evidence is rapidly growing regarding a role of astroglial cells in the pathogenesis of Alzheimer’s disease (AD), and the hippocampus is one of the important brain regions affected in AD. While primary astroglial cultures, both from wild-type mice and from rodent models of AD, have been useful for studying astrocyte-specific alterations, the limited cell number and short primary culture lifetime have limited the use of primary hippocampal astrocytes. To overcome these limitations, we have now established immortalized astroglial cell lines from the hippocampus of 3xTg-AD and wild-type control mice (3Tg-iAstro and WT-iAstro, respectively). Both 3Tg-iAstro and WT-iAstro maintain an astroglial phenotype and markers (glutamine synthetase, aldehyde dehydrogenase 1 family member L1 and aquaporin-4) but display proliferative potential until at least passage 25. Furthermore, these cell lines maintain the potassium inward rectifying (Kir) current and present transcriptional and proteomic profiles compatible with primary astrocytes. Importantly, differences between the 3Tg-iAstro and WT-iAstro cell lines in terms of calcium signaling and in terms of transcriptional changes can be re-conducted to the changes previously reported in primary astroglial cells. To illustrate the versatility of this model we performed shotgun mass spectrometry proteomic analysis and found that proteins related to RNA binding and ribosome are differentially expressed in 3Tg-iAstro vs WT-iAstro. In summary, we present here immortalized hippocampal astrocytes from WT and 3xTg-AD mice that might be a useful model to speed up research on the role of astrocytes in AD.

## Introduction

While in Alzheimer’s disease (AD) astrocytes have been historically associated with reactive gliosis and neuroinflammation, a growing body of evidence suggests that astroglial alterations occur in the early stages of AD, compromising their housekeeping and homeostatic functions that in turn may result in synaptic and neuronal malfunction^1,2^.

Most of the information about the role of astrocytes in brain pathology has been collected in in vitro experiments on primary cultures. Easy to prepare and handle, astroglial primary cultures are made from different brain areas^3,4^ and different animal species^5–8^. However, primary cultures present several limitations such as inter-culture variability, short culture lifetime and limited number of cells if cultures are prepared from specific brain areas, such as the hippocampus. To overcome these limitations, immortalized astroglial lines have been proposed. The first attempts to generate a permanent astroglial cell line, not deriving from brain tumors, date back to early eighties^9^. Since then, a number of immortalized astroglial lines have been established^10–23^. Most of them derive from a highly heterogeneous population of cortical primary astrocytes; however, immortalization of astrocytes from brain regions other than cortex have also been reported, e.g., from the cerebellum^9^ or from the midbrain^21^. Surprisingly, few attempts have been reported to immortalize astrocytes from hippocampus and also from animal models of AD. In this regard, Morikawa et al.^18^ have generated immortalized astrocytes from ApoE2, ApoE3 and ApoE4 knock-in mice.

In this report we introduce immortalized astroglial lines from the hippocampus of a well-characterized AD mouse model, 3xTg-AD, and from wild-type (WT) control mice, from now on referred to as 3Tg-iAstro and WT-iAstro, respectively. WT-iAstro cell lines show features of primary hippocampal astrocytes such as basic electrophysiological properties, and a similar transcriptional and proteomic profile. More importantly, 3Tg-iAstro show alterations in transcription and deregulation of Ca^2+^ signaling as it was reported for its primary counterparts. To illustrate the versatility of this model we also performed shotgun mass spectrometry proteomic analysis and found that proteins related to RNA binding and ribosome are differentially expressed in 3Tg-iAstro vs WT-iAstro. These data demonstrate that iAstro lines represent a versatile and useful cellular model to investigate astroglial AD-related pathobiology.

## Results

### Generation of immortalized hippocampal astrocytes (iAstro) from WT and 3xTg-AD mice

Six immortalized cell lines from WT (WT-iAstro#1–6) and from 3xTg-AD (3Tg-iAstro#1–6) mice were generated, from separate primary astrocyte cell cultures. For immortalization, primary astroglial cultures were first depleted of microglial cells by magnetic-assisted cell sorting (MACS) using anti-CD11b-conjugated microbeads in order to obtain a population of highly purified astrocytes. Astrocytes were then transduced using retrovirus expressing SV40 large T antigen. Transformed cells were selected in G418, amplified and stabilized for 12 passages prior to characterization. No clonal selection was performed to maintain the natural hererogeneity of the cultures.

For logistic and experimental setting convenience, four lines for each strain were characterized for morphology and astroglial marker expression. The other two cell lines were confirmed for morphological identity to other lines, but were maintained as backups and have not been characterized (#1 and #5 for WT-iAstro and #1 and #4 for 3Tg-iAstro lines).

**Fig. 1:**
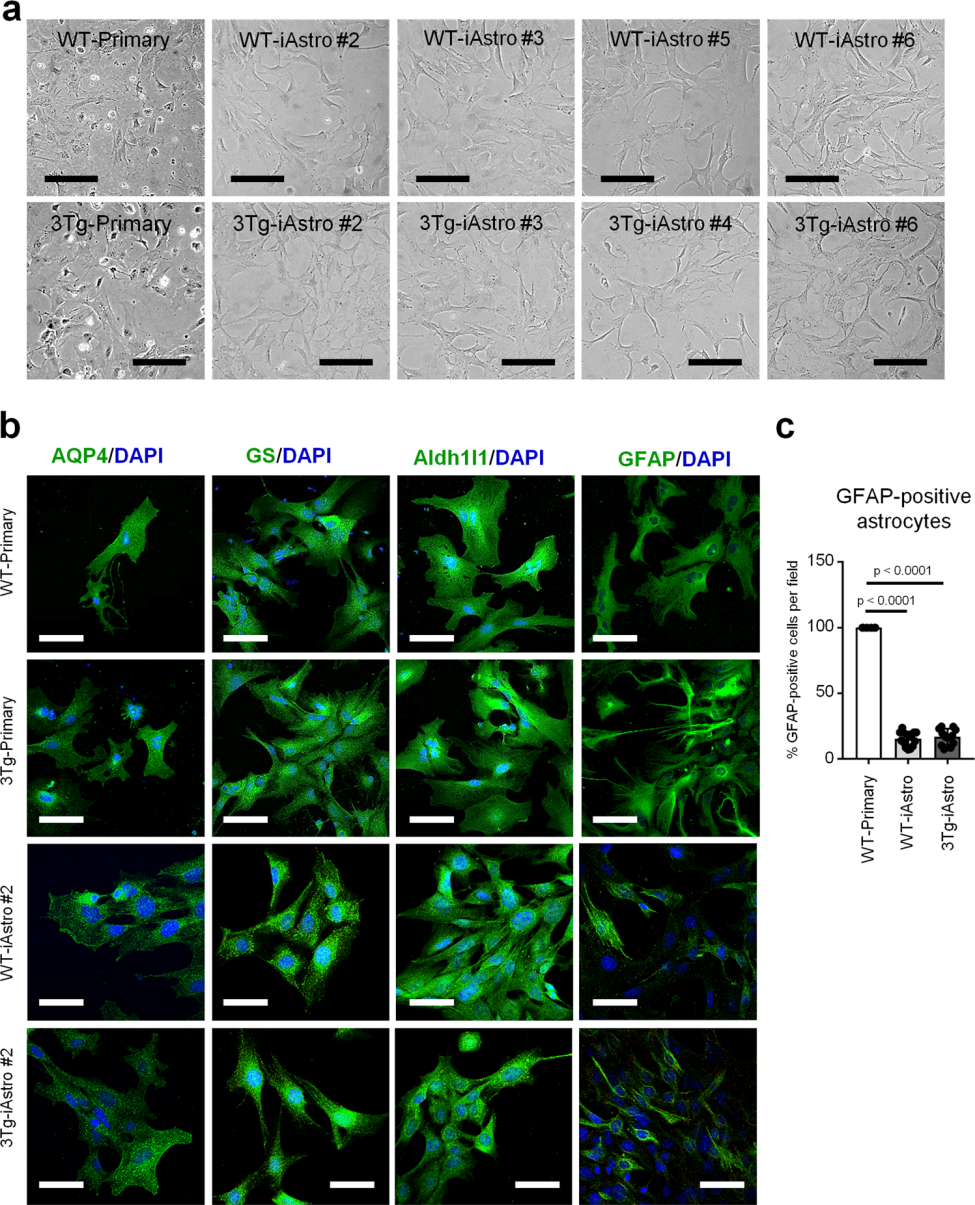
Characterization of WT- and 3Tg-iAstro lines. **a** Phase contrast images of four WT-iAstro and four 3Tg-iAstro lines at passage 15. Bar, 100 μm. **b** Immunofluorescence images of WT-iAstro#2 and 3Tg-iAstro#2, stained with anti-AQP4, anti-GS, anti-Aldh1l1 and anti-GFAP antibodies. Bar, 50 μm. The images shown in (**a**, **b**) are representative of *n* = 3 independent experiments. **c** Quantification of GFAP-positive cells in WT-iAstro#2 and 3Tg-iAstro#2 lines. Data expressed as mean ± SD % of 15 fields of GFAP-positive cells evaluating a total of 359 WT- and 514 3Tg-iAstro cells. In (**b**, **c**), other characterized independently generated iAstro lines show similar results in immunostaining of astroglial markers and in quantification of GFAP-positive cells (data not shown)

iAstro cells show a morphology similar and virtually indistinguishable to those of primary hippocampal astrocytes in bright field microscopy (Fig. 1a). We also evaluated the expression of the astroglial markers aquaporin-4 (AQP4), glutamine synthetase (GS) and aldehyde dehydrogenase 1 family member l1 (Aldh1l1) (Fig. 1b) by immunocytochemistry. Importantly, we found expression of all three markers in all cell lines. Immunocytochemical analysis for glial fibrillary acidic protein (GFAP) showed, instead, that only a small proportion of cells were positive in the established immortalized cell lines (15.4 ± 5.3 % in WT-iAstro vs 16.7 ± 5.9 % in 3Tg-iAstro cells, *p* > 0.05) (Fig. 1c), while 100% of cells were GFAP positive in WT- and 3Tg-primary astrocytes.

**Fig. 2:**
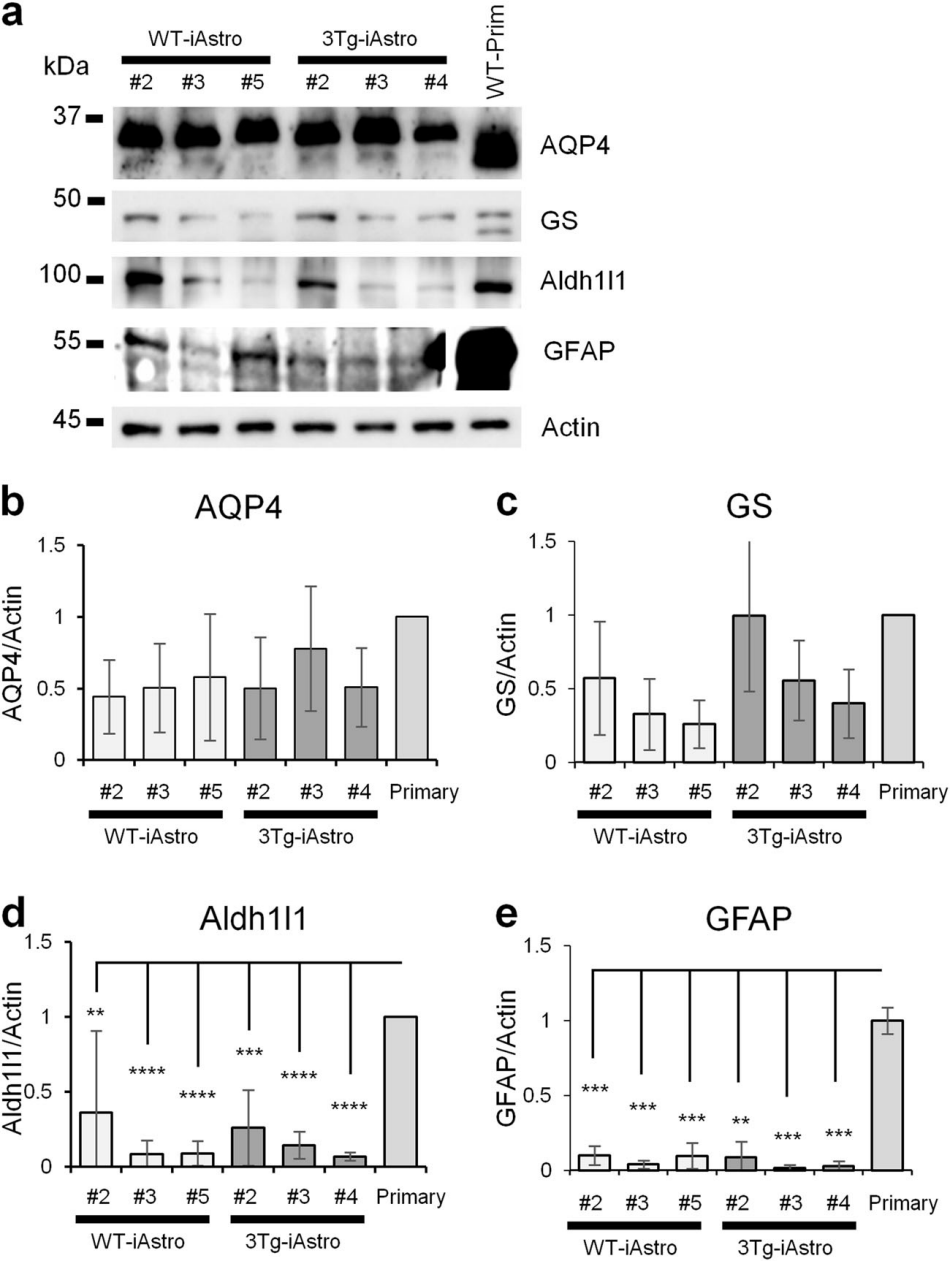
Western blot analysis and quantification of astroglial marker proteins Aqp4, GS, Aldh1l1 and GFAP. Western blot analysis (**a**) was performed from four independently generated iAstro lines for each genotype (WT-iAstro#2, #3, #5 and #6, and 3Tg-iAstro#2, #3, #4 and #6). Each point represents mean ± SEM of 3 independent experiments. Actin was used as loading control. ANOVA followed by Tukey’s post-hoc test was used for statistical analysis. For Aqp4 (**b**) and GS (**c**) there were no significant differences. For Aldh1l1 (**d**) and GFAP (**e**) the differences were significant for iAstro lines vs primary astrocytes: **p < 0.01; ***p < 0.001; ****p < 0.0001

We also decided to have a quantitative measure of the expression of the three markers by evaluating protein levels in western blotting on three of the four cell lines. The three markers were mostly decreased compared to primary cell lines, albeit at different levels. AQP4 was the least decreased, while Aldh1l1 was the most decreased, with qualitative consistency in the immortalized cell lines tested. Importantly, not only were all the markers detectable, but they were also represented at similar levels between the WT- and 3Tg-iAstro cell lines (Fig. 2).

We also evaluated the ability of the iAstro cell lines to be passaged in culture. iAstro lines did not change their morphology or marker expression significantly at least up to 20th passage (not shown).

### Kir currents are present but are not different in WT-iAstro and 3Tg-iAstro

**Fig. 3:**
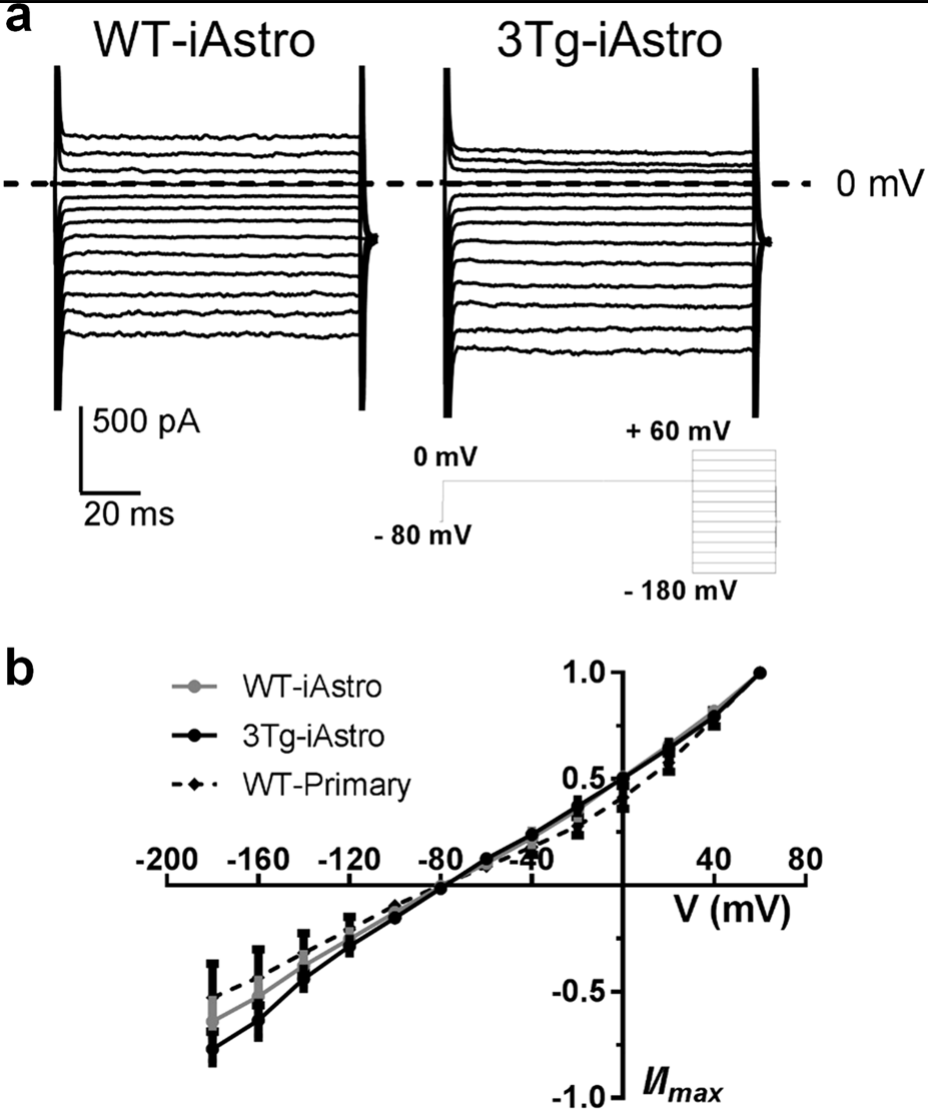
Kir are functional in both WT- and 3Tg-iAstro lines. **a** Representative Kir currents elicited in WT-iAstro#2 and 3Tg-iAstro#2 astrocytes in basal conditions. Inset shows the voltage step protocol applied (from −180 mV to +60 mV for 100 ms in 20 mV increments). Voltage at 0 mV is shown (dotted line). **b** Current/voltage relationship (I/V curve) elicited in WT-primary (black dotted line), WT-iAstro#2 (gray line) and 3Tg-iAstro#2 (black line) astrocytes. Raw current (I) values were normalized to maximum I obtained at +60 mV for each cell. Each point is shown as average ± SEM (*n* = 6)

Maintenance of ionic balance is one of the housekeeping functions of astrocytes and a feature which might have been lost during immortalization. Potassium buffering by inwardly rectifying K^+^ (Kir) channels during neuronal activity is fundamental to maintain adequate synaptic transmission and neuronal excitability^24^. We have previously shown that Kir channels are functionally expressed in primary hippocampal astrocytes^25^. Therefore, we performed patch-clamp experiments in WT-iAstro#2 and 3Tg-iAstro#2 lines. To confirm that Kir function was not affected by the immortalization process, control hippocampal mouse astrocytes were also patched in order to record Kir current. Cells were exposed to a step protocol of increasing voltage (20 mV increments) from −180 mV to +60 mV to record current elicited by Kir channels. Before each voltage step increment, cells were kept at 0 mV for 300 ms in order to block outward potassium flow^26^. Application of this protocol triggered Kir current in the cell lines tested (Fig. 3a). Current (I), measured at each step of recording protocol, was plotted over corresponding applied voltage (V) to determine I/V curve for Kir channels in both cell lines. Raw current values were normalized to maximum I obtained at +60 mV for each cell (Fig. 3b). No differences were found in iAstro Kir currents through at least five passages. No significant differences were observed in Kir currents between control primary astrocytes and WT- and 3Tg-iAstro lines.

### iAstro cells are capable of glutamate uptake

Another fundamental function of astrocytes in the regulation of synaptic transmission is the uptake of glutamate through the action of sodium-dependent glutamate transporters^27^. In the hippocampus, GLT-1 (excitatory amino acid transporter 2 (EAAT2)) is the major glutamate transporter^28^. Therefore, we investigated the expression of GLT-1 and glutamate uptake in iAstro lines. As shown in Supplementary Figure 1A, anti-GLT-1 staining revealed membrane-localized expression of GLT-1 in both primary astrocytes and in iAstro lines. Supplementary Figure 1B shows that WT-iAstro cells are capable of glutamate uptake from the medium with a rate (10.73 ± 1.19 μmol/g protein, *n* = 3) comparable to that of primary astrocytes (19.38 ± 3.74 μmol/g protein, *n* = 3; *p* = 0.092). No differences were found in glutamate uptake between WT-iAstro and 3Tg-iAstro lines (10.77 ± 2.14 μmol/g protein, *n* = 3; *p* = 0.12).

### ATP-induced Ca^2+^ signaling is altered in immortalized hippocampal astrocytes from 3xTg-AD mice

Astrocytes respond to external stimuli primarily by generating intracellular Ca^2+^ signals, employing a combination of release of Ca^2+^ from the internal stores via metabotropic mechanisms and Ca^2+^ entry from the extracellular space through the plasma membrane via store-operated Ca^2+^ entry^29–31^. We have previously shown that astrocytic Ca^2+^signaling in AD is altered using a variety of protocols/models usually employed for neuronal evaluation, including Aβ oligomer treatment^32^, an uncoupling peptide of ADAM10 and SAP97^33^, or triple transgenic mice^34^. Astrocytic Ca^2+^ signaling has been shown to be altered in AD also by others in vivo and in vitro^35–38^.

**Fig. 4:**
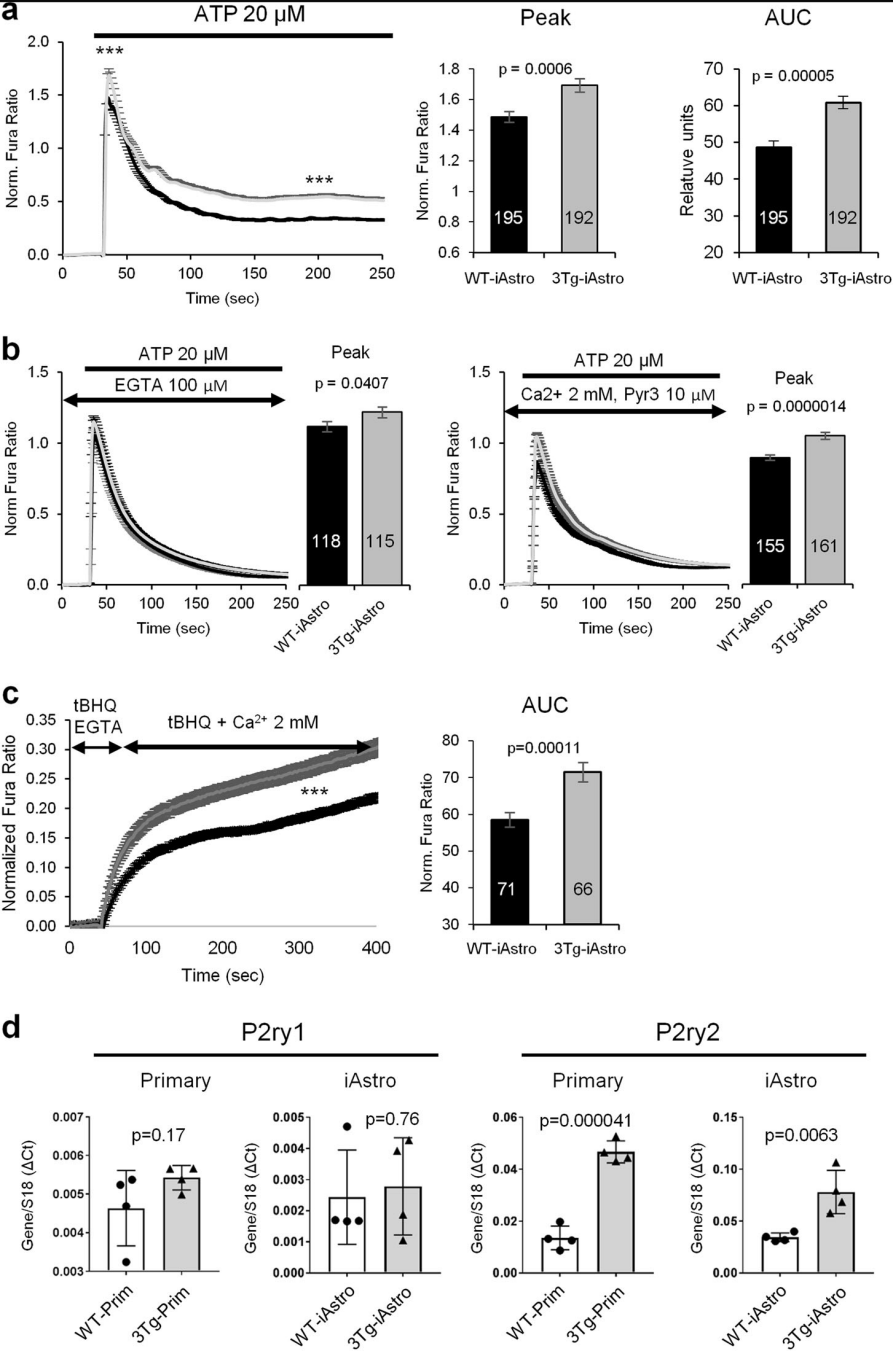
Calcium signaling alterations in 3Tg-iAstro as compared to WT-iAstro lines. **a** Fura-2-loaded WT- and 3Tg-iAstro cells were stimulated with 20 μM ATP in Ca^2+^-containing KRB solution. Data are expressed as mean of peak ± SEM or mean of area under the curve (AUC) ± SEM of 195 cells (WT-iAstro#2; 1.39 ± 0.035 norm. Fura ratio) and 192 cells (3Tg-iAstro#2; 1.69 ± 0.045 norm. Fura ratio, *p* = 0.0006) from 12 coverslips from 3 independent experiments. ***p < 0.001. **b** WT- and 3Tg-iAstro cells were stimulated with 20 μM ATP in Ca^2+^-free KRB solution (left panel; mean of peak ± SEM of 118 cells (WT-iAstro#2; 1.19 ± 0.03 norm. Fura ratio) and 115 cells (3Tg-iAstro#2; 1.31 ± 0.039 norm. Fura ratio, *p* = 0,040) or in Ca^2+^-containing KRB supplemented with 10 μM Pyr3, a store-operated Ca^2+^ channel blocker (right panel; mean of peak ± SEM of 155 cells (WT-iAstro#2; 0.89 ± 0.018 norm. Fura ratio) and 161 cells (3Tg-iAstro#2; 1.05 ± 0.025 norm. Fura ratio, *p* = 0.000001). **c** Fura-2-loaded cells were depleted of Ca^2+^ with 20 μM tBHQ (a SERCA blocker) in Ca^2+^-free KRB for 5 min after which Ca^2+^ was re-added and Fura-2 ratio was measured. Data are expressed as mean ± SEM of AUC of 71 WT-iAstro#2 cells (58.51 ± 1.94 AUC of norm. Fura ratio) and 66 3Tg-iAstro#2 cells (71.5 ± 1.64 AUC of norm. Fura ratio, *p* = 0.00011) from 8 coverslips from 3 independent experiments. **d** Real-time PCR of P2ry1 (left graphs) and P2ry2 (right graph) from primary astrocytes and iAstro lines. Values represent mean ± SD ΔC(t) of gene/S18 of four independent cultures. Unpaired two-tailed Student’s *t*-test was used for statistical analysis

To validate our immortalized cell lines, we therefore decided to investigate astrocytic Ca^2+^ signaling using Fura-2 single cell Ca^2+^ imaging. In pilot experiments, 4 WT lines (WT-iAstro#2, #3, #5 and #6) and 4 3Tg-iAstro lines (3Tg-iAstro#2, #3, #4 and #6) were tested for adenosine triphosphate (ATP; 20 μM)-induced Ca^2+^ responses, which is among the signaling pathways found altered in AD^34^. As no significant differences were observed between lines of the same genotype (data not shown), WT-iAstro#2 and 3Tg-iAstro#2 were chosen for further analysis. Traces and histograms in Fig. 4a represent mean ± SEM of 195 cells from WT-iAstro (1.39 ± 0.035 norm. Fura ratio) and 192 cells from 3Tg-iAstro lines (1.69 ± 0.045 norm. Fura ratio) and show that 3Tg-iAstro exhibited significantly higher amplitude of Ca^2+^ responses (*p* = 0.0006). As it can be observed, application of ATP to iAstro produces a long-lasting “after-peak” shoulder which is significantly higher in 3Tg-iAstro than in WT-iAstro (48.58 ± 1.86 area under the curve (AUC) of norm. Fura ratio for WT-iAstro vs 60.83 ± 1.71 for 3Tg-iAstro, *p* = 0.00005) (Fig. 4c). Given that in primary astrocytes we had observed a similar effect with mGluR5 stimulation that was mediated by store-operated Ca^2+^ entry (SOCE)^39^, we decided to further explore the effect of ATP. Applications of ATP (20 μM) in Ca^2+^-free solution or pre-treatment with the SOCE inhibitor Pyr3 (10 μM) completely eliminated the shoulder in both WT-iAstro and 3Tg-iAstro, while the amplitudes of the responses were higher in 3Tg-iAstro cells compared to WT-iAstros (1.31 ± 0.039 norm. Fura ratio, *n* = 115, for 3Tg-iAstro vs 1.19 ± 0.03, *n* = 118, for WT-iAstro, *p* = 0.040 in Ca^2+^-free solution; 1.05 ± 0.025 norm. Fura ratio, *n* = 161, for 3Tg-iAstro vs 0.89 ± 0.018, *n* = 155, for WT-iAstro, *p* = 0.000001 in Pyr3-treated cells) (Fig. 4b). This suggests that ATP, similarly to (*RS*)-3,5-Dihydroxyphenylglycine (DHPG, an agonist of group I metabotropic glutamate receptors), induced SOCE-mediated Ca^2+^ entry through the plasma membrane. This was confirmed by re-addition of Ca^2+^ after depletion of the endoplasmic reticulum Ca^2+^ stores with a SERCA inhibitor (tBHQ, 20 μM, 5 min) in a Ca^2+^-free Krebs-Ringer modified buffer (KRB) solution (71.5 ± 1.64 AUC of norm. Fura ratio, *n* = 66, for 3Tg-iAstro vs 58.51 ± 1.94, *n* = 71, for WT-iAstro, *p* = 0.00011) (Fig. 4c).

In the current experimental setting the astroglial Ca^2+^ signals are likely to be mediated by two types of purinergic receptors, P2Y1 and P2Y2. Relative quantification of messenger RNA (mRNA) of P2ry1 and P2ry2 in WT and 3Tg-iAstro lines using real-time PCR showed a higher levels of P2ry2 mRNA compared with P2ry1; moreover, P2ry2 was significantly more expressed in 3Tg-iAstro compared with WT, while levels of P2ry1 were not different. These data suggest that P2Y2 but not P2Y1 may mediate enhanced sensitivity of 3Tg-iAstro to ATP (Fig. 4d).

Surprisingly, and contrary to our previous observations on primary astrocytes^34^, we found that iAstro cell lines did not respond to DHPG.

### iAstro lines maintain gene expression differences found previously between WT and 3xTg-AD primary astrocytes

We recently performed a whole-genome microarray analysis of purified primary hippocampal astrocytes^40^ in which bioinformatic analysis revealed expression differences between astrocytes prepared from WT and from 3Tg animals. To further characterize our immortalized cell lines we therefore primed 14 of the identified genes that showed modifications (Car9, P2ry2, Padi2, Col2a1, Col4a6, Fbln2, Cacna2d3, Panx1, Pcdh17, Trim12, Ccl27a PESKY, Ptchd2, Slc2a4 and Samd4). This set was composed of 6 genes (Pcdh17, Col2a1, Col4a6, Fbln2, Cacna2d3, Panx1) involved in cell adhesion (a gene ontology cluster that was found over-represented), two down-regulated genes (Padi2 and Samd4) and a set of 6 up-regulated genes (Trim12, Ptchd2, Slc2a4, Ccl27a PESKY, Car9 and P2ry2).

**Fig. 5:**
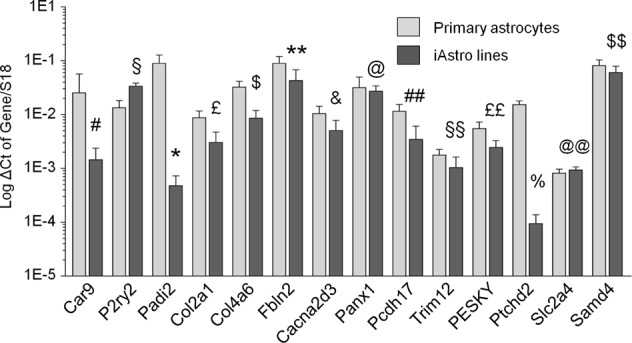
Comparison of transcriptional levels of selected genes between primary astrocyte cultures and WT-iAstro cells. The graph shows log scale of RNA levels for specific genes detected in primary mouse hippocampal astrocytes (light gray bars) and in four WT-iAstro lines (#2, #3, #5, #6) (dark gray bars). Data are expressed as mean ± SD ΔC(t) of gene/S18 of four independent cultures. Unpaired two-tailed Student’s *t*-test was used for statistical analysis; ^#^*p* = 0.17; ^§^*p* = 0.00054; *p = 0.0028; ^£^*p* = 0.012; ^$^*p* = 0.0019; **p = 0.048; ^&^*p* = 0.055; ^@^*p* = 0.064; ^##^*p* = 0.013; ^§§^*p* = 0.091;^££^*p* = 0.019; ^%^*p* = 0.00002; ^@@^*p* = 0.021; ^$$^*p* = 0.19

First, we compared levels of the genes between primary WT astrocytes and WT-iAstro, comparing ΔC(t) of a gene of interest with respect to S18 C(t). Given the low variability between different primary cultures and between different iAstro cultures, comparison was made averaging data from WT-iAstro #2, #3, #5 and #6 and four primary cultures. As shown in Fig. 5, most of the genes were expressed in the same order of magnitude between primary and immortalized cells, although a few genes (Car9, Padi2 and Ptchd2) showed a reduced expression of at least one order of magnitude differences.

**Fig. 6:**
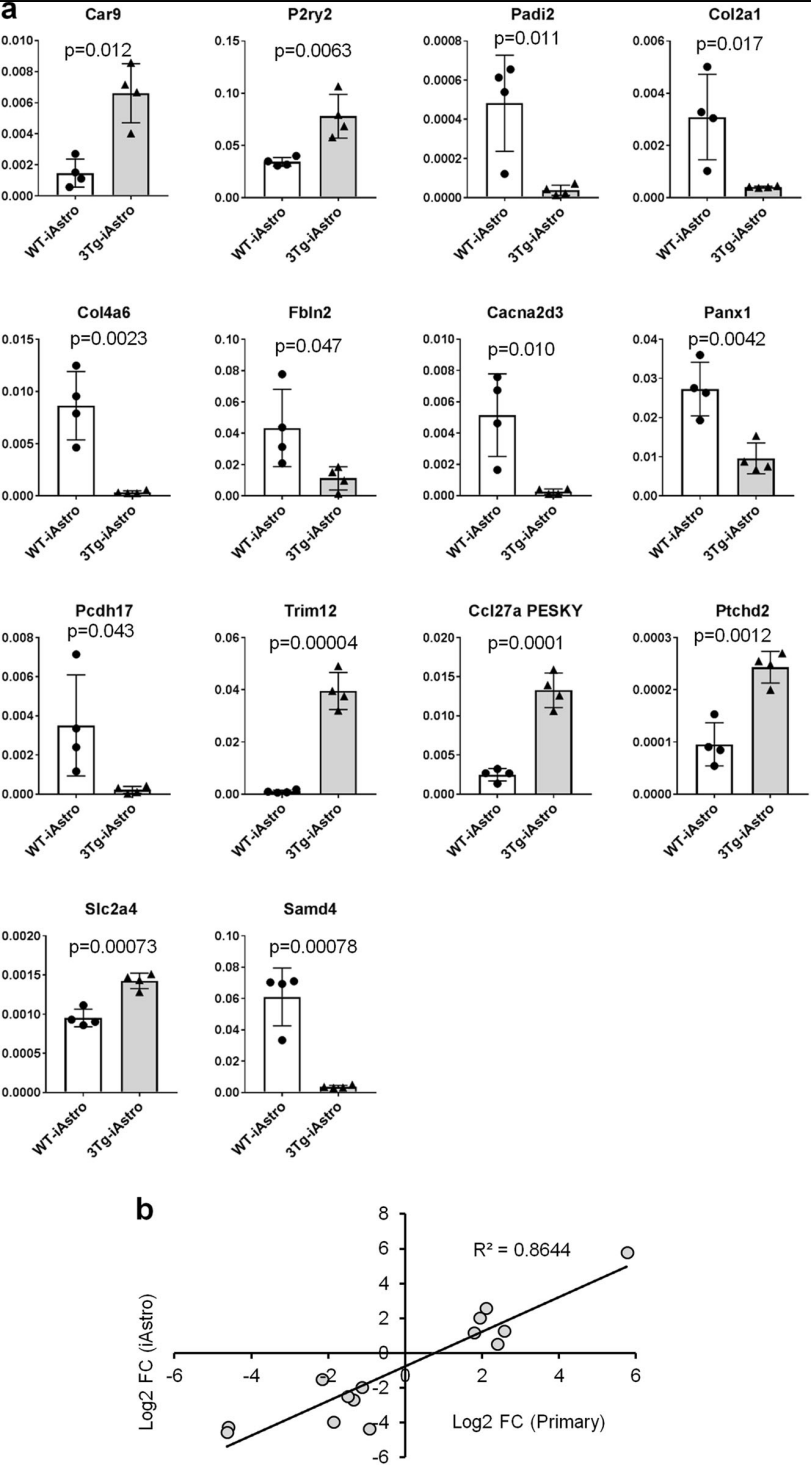
The differences between primary cultured astrocytes from WT and 3xTg mice are retained in the iAstro cell lines. **a** RT-PCR performed on samples prepared from WT-iAstro#2, #3, #5, #6 and 3Tg-iAstro#2, #3, #4, #6. Data are expressed as mean ± SD ΔCt of gene/S18 of runs performed in triplicate. Unpaired two-tailed Student’s *t*-test was used for statistical analysis. **b** Correlation plot of differential gene expression between primary astrocytes and iAstro lines. Abscissa axis shows log2 (fold change) of 3xTg-AD primary cultured hippocampal astrocytes vs WT astrocytes. Ordinate axis shows log2(fold change) of 3Tg-iAstro vs WT-iAstro lines

Our aim, though, was to validate, independently of the expression levels, whether the differences in expression between primary WT and 3Tg astrocytes could be recapitulated in the immortalized cell lines. As shown in Fig. 6a, all 14 genes followed the changes found previously in primary astrocytes^40^. A plot in Fig. 6b shows high degree of correlation between primary and immortalized astrocytes. Taken together, these results show that after the immortalization hippocampal astrocytes retain the transcriptional changes found in primary 3xTg-AD vs WT astrocytes for, at least, a selected group of genes.

### Immortalized astrocytes up-regulate iNOS in response to pro-inflammatory stimuli

The role of astrocytes in neuroinflammation is well ascertained^31,41^, and therefore we have investigated if iAstro lines responded to pro-inflammatory stimuli like bacterial lipopolysaccharide (LPS) or tumor necrosis factor-α (TNFα). As shown in Supplementary Figure 2, inducible nitric oxide synthase (iNOS) was induced upon treatment with both LPS (100 ng/ml for 3 h) and TNFα treatment (20 ng/ml for 6 h). No differences were found between WT and 3Tg-primary astrocytes or iAstro lines.

We recently reported that transforming growth factor-β2 (TGFβ2) and TGFβ3 were up-regulated at mRNA levels in 3Tg-primary astrocytes as compared to WT astroglial cultures^42^. We, therefore, investigated the levels of mRNA of TGFβ2 and TGFβ3 in both non-stimulated and LPS-treated iAstro lines. As shown in Supplementary Figure 3, in line with cultured primary astrocytes^42^, TGFβ3 is the most expressed TGFβ isoform in iAstro lines. Moreover, TGFβ3 was significantly up-regulated in 3Tg-iAstro (0.267 ± 0.02 ΔC(t) TGFβ3/S18, *n* = 4) compared to WT-iAsro cells (0.094 ± 0.014 ΔC(t) TGFβ3/S18, *n* = 4; *p* = 0.0012) (Supplementary Figure 3B). No differences were found in TGFβ2 or TGFβ3 mRNA levels upon treatment with LPS.

### Mass spectrometry proteomics revealed alterations in translation and ribosome in 3Tg-iAstro compared to WT-iAstro

**Fig. 7:**
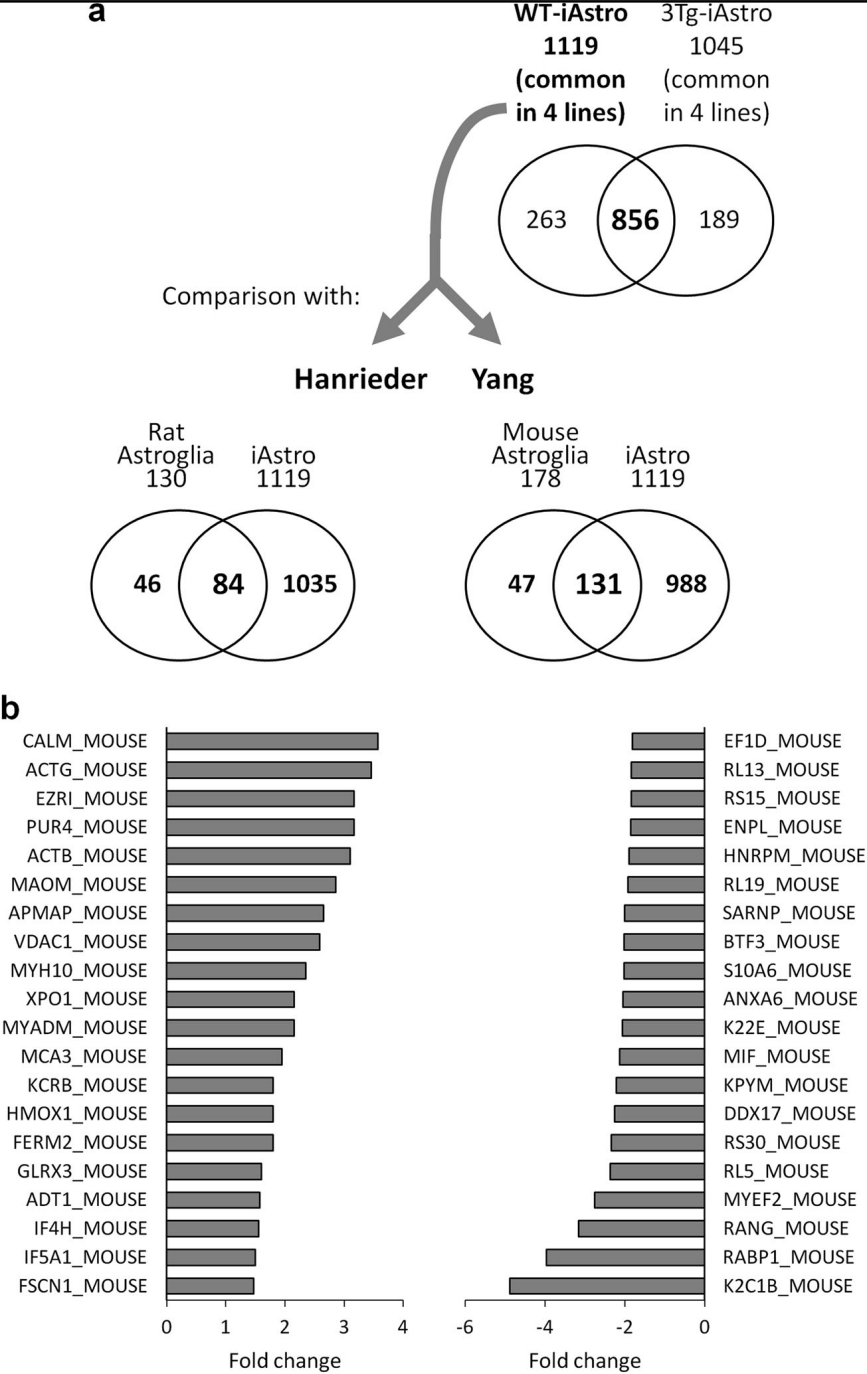
Mass spectrometry proteomics of differentially expressed proteins. **a** Protein lysates of WT-iAstro#2, #3, #5, #6 and 3Tg-iAstro#2, #3, #4, #6 were subjected to LC-MS/MS with consequent SWATH-MS (see details in Methods section). In total, 1119 and 1045 proteins were detected in WT- and 3Tg-iAstro lines, respectively, among which 856 were expressed in both types of samples. The list of 1119 proteins common for all four WT-iAstro lines was compared for presence of common entries with two astroglial proteomics datasets contributed by Hanreider et al.^43^ and Yang et al.^44^. **b** Top 20 of up- and down-regulated proteins emerged from mass spectrometry proteomics

To further evaluate the iAstro lines, we performed a shotgun mass spectrometry proteomic analysis using the four WT and four 3Tg-iAstro lines. In total, 1119 and 1045 proteins were identified, common to the four analyzed WT- and 3Tg-iAstro lines, respectively (Supplementary Table 1), of which 856 proteins were present in both WT- and 3Tg-iAstro lines (Fig. 7a).

To demonstrate that WT-iAstro cells retain the astrocytic phenotype we compared their proteomic profile with two published datasets obtained through matrix-assisted laser desorption/ionization time-of-flight (MALDI-TOF) mass spectrometry. The first one, provided by Hanrieder et al.^43^, featured 130 unambiguous identified proteins from cultured rat cortical neuroglia. Our dataset included 84 out of 130 (64.6%) of their entries (see Supplementary Table 2A). Among them, cytochrome C oxidase (COX5A), cytochrome C (CYC), ubiquitin (RS27A), chaperonin 10 (CH10), macrophage inhibitor factor (MIF), acetyl co-A binding protein (ACBP), thioredoxin (THIO), calmodulin (CALM), thymosin beta-10 (TYB4) and ribosomal protein S28 (RS28), reported by Hanrieder et al.^43^ as the most astrocyte-specific proteins compared to both oligodendrocytes and microglia, were notably all present in our list. Secondly, we compared iAstro proteins with the proteomic profile provided by Yang et al.^44^, consisting of 178 different proteins from mouse cortical cultured astrocytes. Our list covered 131 out of 178 proteins (73.6%) from the dataset provided by Yang et al.^44^ (see Fig. 7a and Supplementary Table 2B). Note a relatively small number of detected proteins in Hanrieder et al.^43^ and Yang et al.^44^ datasets compare to our list of proteins. This may be due to differences in the workflow of the proteome analysis.

Next, we performed differential gene expression analysis and identified 73 proteins (*p* < 0.05; cut-off 30% fold change) that were significantly changed between WT-iAstro and 3Tg-iAstro, of which 23 were up-regulated and 50 were down-regulated (Fig. 7b, Table 1 and Supplementary Table 3). Functional classification Gene Ontology (GO) analysis of all 73 differentially expressed proteins using DAVID (Database for Annotation, Visualization and Integrated Discovery) online GO tool returned two groups with significant enrichment score: the first group (enrichment score 12.8) contained 11 proteins all of which were components of ribosome; the second group (enrichment score 8.9) was composed of 5 proteins related to RNA binding and to the formation of ribonucleoprotein complex (Supplementary Table 4A). To explore functional significance of up-regulated vs down-regulated proteins, we analyzed separately 23 up-regulated and 50 down-regulated proteins. Analysis of up-regulated proteins did not return significantly overrepresented GO terms, while functional annotation GO analysis of the 50 down-regulated proteins returned 10 significantly overrepresented GO terms which were related to RNA binding, ribonucleoprotein complex, ribosome and nucleus (Table 2 and Supplementary Table 4B).

**Table 1:** Top 20 up- and down-regulated proteins in 3Tg-iAstro compared to WT-iAstro lines

Uniprot_ID	Description	Fold change	*P* value
Top 20 of UP-regulated proteins			
CALM_MOUSE	Calmodulin OS = Mus musculus GN = Calm1 PE = 1 SV = 2	3.57	0.00067
ACTG_MOUSE	Actin, cytoplasmic 2 OS = Mus musculus GN = Actg1 PE = 1 SV = 1	3.46	0.00229
EZRI_MOUSE	Ezrin OS = Mus musculus GN = Ezr PE = 1 SV = 3	3.16	0.00189
PUR4_MOUSE	Phosphoribosylformylglycinamidine synthase OS = Mus musculus GN = Pfas PE = 2 SV = 1	3.16	0.00287
ACTB_MOUSE	Actin, cytoplasmic 1 OS = Mus musculus GN = Actb PE = 1 SV = 1	3.10	0.00358
MAOM_MOUSE	NAD-dependent malic enzyme, mitochondrial OS = Mus musculus GN = Me2 PE = 2 SV = 1	2.86	0.01149
APMAP_MOUSE	Adipocyte plasma membrane-associated protein OS = Mus musculus GN = Apmap PE = 1 SV = 1	2.66	0.04597
VDAC1_MOUSE	Isoform Mt-VDAC1 of Voltage-dependent anion-selective channel protein 1 OS = Mus musculus GN = Vdac1	2.58	0.04867
MYH10_MOUSE	Myosin-10 OS = Mus musculus GN = Myh10 PE = 1 SV = 2	2.35	0.01125
XPO1_MOUSE	Exportin-1 OS = Mus musculus GN = Xpo1 PE = 1 SV = 1	2.16	0.01711
MYADM_MOUSE	Myeloid-associated differentiation marker OS = Mus musculus GN = Myadm PE = 2 SV = 2	2.15	0.03671
MCA3_MOUSE	Eukaryotic translation elongation factor 1 epsilon-1 OS = Mus musculus GN = Eef1e1 PE = 2 SV = 1	1.95	0.03302
KCRB_MOUSE	Creatine kinase B-type OS = Mus musculus GN = Ckb PE = 1 SV = 1	1.80	0.04875
HMOX1_MOUSE	Heme oxygenase 1 OS = Mus musculus GN = Hmox1 PE = 1 SV = 1	1.80	0.01236
FERM2_MOUSE	Fermitin family homolog 2 OS = Mus musculus GN = Fermt2 PE = 1 SV = 1	1.80	0.02534
GLRX3_MOUSE	Glutaredoxin-3 OS = Mus musculus GN = Glrx3 PE = 1 SV = 1	1.60	0.03293
ADT1_MOUSE	ADP/ATP translocase 1 OS = Mus musculus GN = Slc25a4 PE = 1 SV = 4	1.58	0.00764
IF4H_MOUSE	Eukaryotic translation initiation factor 4H OS = Mus musculus GN = Eif4h PE = 1 SV = 3	1.55	0.00594
IF5A1_MOUSE	Eukaryotic translation initiation factor 5A-1 OS = Mus musculus GN = Eif5a PE = 1 SV = 2	1.50	0.03688
FSCN1_MOUSE	Fascin OS = Mus musculus GN = Fscn1 PE = 1 SV = 4	1.47	0.02807
Top 20 of DOWN-regulated proteins			
K2C1B_MOUSE	Keratin, type II cytoskeletal 1b OS = Mus musculus GN = Krt77 PE = 1 SV = 1	−4.89	0.02491
RABP1_MOUSE	Cellular retinoic acid-binding protein 1 OS = Mus musculus GN = Crabp1 PE = 1 SV = 2	−3.96	0.00811
RANG_MOUSE	Ran-specific GTPase-activating protein OS = Mus musculus GN = Ranbp1 PE = 1 SV = 2	−3.15	0.000058
MYEF2_MOUSE	Myelin expression factor 2 OS = Mus musculus GN = Myef2 PE = 1 SV = 1	−2.76	0.00512
RL5_MOUSE	60S ribosomal protein L5 OS = Mus musculus GN = Rpl5 PE = 1 SV = 3	−2.36	0.00058
RS30_MOUSE	40S ribosomal protein S30 OS = Mus musculus GN = Fau PE = 1 SV = 1	−2.33	0.01976
DDX17_MOUSE	Probable ATP-dependent RNA helicase DDX17 OS = Mus musculus GN = Ddx17 PE = 1 SV = 1	−2.25	0.0443
KPYM_MOUSE	Isoform M1 of pyruvate kinase PKM OS = Mus musculus GN = Pkm	−2.21	0.01086
MIF_MOUSE	Macrophage migration inhibitory factor OS = Mus musculus GN = Mif PE = 1 SV = 2	−2.13	0.04215
K22E_MOUSE	Keratin, type II cytoskeletal 2 epidermal OS = Mus musculus GN = Krt2 PE = 1 SV = 1	−2.06	0.04617
ANXA6_MOUSE	Annexin A6 OS = Mus musculus GN = Anxa6 PE = 1 SV = 3	−2.05	0.01972
S10A6_MOUSE	Protein S100-A6 OS = Mus musculus GN = S100a6 PE = 1 SV = 3	−2.02	0.00021
BTF3_MOUSE	Isoform 2 of transcription factor BTF3 OS = Mus musculus GN = Btf3	−2.02	0.00144
SARNP_MOUSE	SAP domain-containing ribonucleoprotein OS = Mus musculus GN = Sarnp PE = 1 SV = 3	−2.01	0.03162
RL19_MOUSE	60S ribosomal protein L19 OS = Mus musculus GN = Rpl19 PE = 1 SV = 1	−1.92	0.0261
HNRPM_MOUSE	Heterogeneous nuclear ribonucleoprotein M OS = Mus musculus GN = Hnrnpm PE = 1 SV = 3	−1.89	0.00045
ENPL_MOUSE	Endoplasmin OS = Mus musculus GN = Hsp90b1 PE = 1 SV = 2	−1.85	0.01527
RS15_MOUSE	40S ribosomal protein S15 OS = Mus musculus GN = Rps15 PE = 2 SV = 2	−1.84	0.00744
RL13_MOUSE	60S ribosomal protein L13 OS = Mus musculus GN = Rpl13 PE = 2 SV = 3	−1.83	0.03011
EF1D_MOUSE	Elongation factor 1-delta OS = Mus musculus GN = Eef1d PE = 1 SV = 3	−1.80	0.03256

**Table 2:** Over-represented GO terms

GO category	GO term	Count	Fold enrichment	Benjamini
GOTERM_CC_DIRECT	GO:0030529~intracellular ribonucleoprotein complex	17	3.30	0.0013
GOTERM_CC_DIRECT	GO:0005840~ribosome	12	3.96	0.0051
GOTERM_CC_DIRECT	GO:0005634~nucleus	37	1.51	0.0121
GOTERM_CC_DIRECT	GO:0022625~cytosolic large ribosomal subunit	8	4.42	0.0391
GOTERM_MF_DIRECT	GO:0044822~poly(A) RNA binding	31	2.25	0.0001
GOTERM_MF_DIRECT	GO:0003735~structural constituent of ribosome	12	3.54	0.0191
GOTERM_MF_DIRECT	GO:0003723~RNA binding	16	2.37	0.0577
KEGG_PATHWAY	mmu03010:Ribosome	12	3.56	0.0065
UP_KEYWORDS	Ribonucleoprotein	15	3.03	0.0074
UP_KEYWORDS	Ribosomal protein	12	3.77	0.0146

**Fig. 8:**
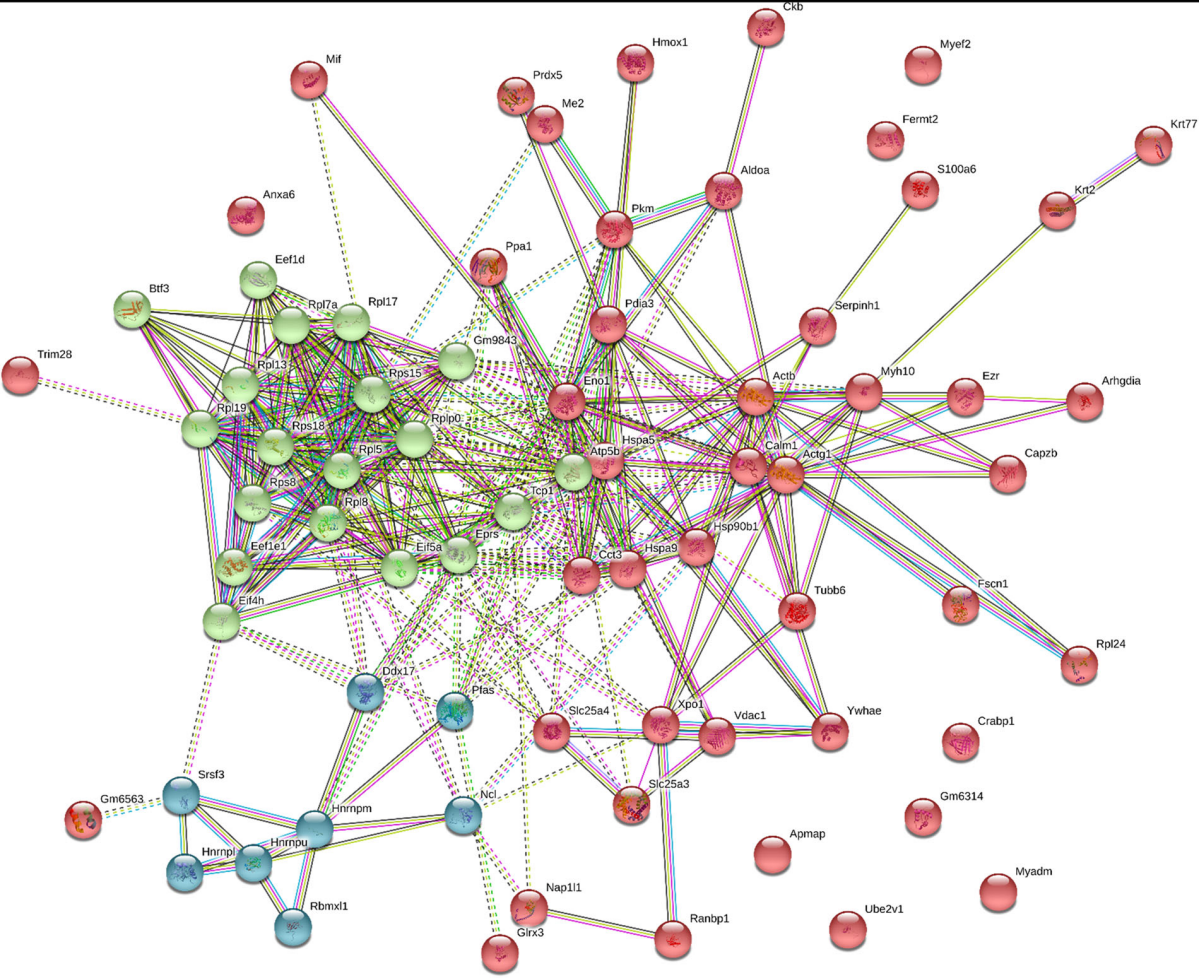
Analysis of possible protein–protein interaction network using STRING tool. List of differentially expressed proteins was subjected to STRING analysis which found significantly more associations between proteins that would have occurred by chance (*p* = 1.0e−16). Three clusters detected by *k*-means algorithm are colored as follows: cluster 1 (46 proteins, red), proteins related to myelin sheet and extracellular exosomes; cluster 2 (19 proteins, green), proteins related to translation and ribosome; cluster 3 (8 proteins, blue), proteins related to nuclear ribonucleoprotein complex and splicing. For related lists of proteins see also Supplementary Table 5

We then used STRING (Search Tool for the Retrieval of Interacting Genes/Proteins) database that allows prediction of protein–protein interacting networks and clustering. For this we used a list of 73 differentially expressed proteins which included both down- and up-regulated hits. As shown in Fig. 8, STRING software found significantly more interactions than may be expected by chance (*p* < 1.0e−16). To search for possible interacting clusters we used STRING *k*-means clustering function followed by GO analysis. Figure 8 shows three clusters found by STRING. GO analysis revealed that cluster #1 (red in Fig. 8) returned two GO terms, *myelin sheath* and *extracellular exosome*. Cluster #2 (green in Fig. 8) returned 13 GO terms related to translation, ribosome and RNA binding, while cluster #3 (blue in Fig. 8) returned 15 GO terms related to nuclear ribonucleoprotein complex and splicing (Supplementary Table 5). Altogether, this analysis suggests that in 3Tg-iAstro lines, the protein synthesis machinery may be impaired.

## Discussion

Here we report the generation and characterization of novel immortalized astroglial lines from the hippocampus of a common AD mouse model, 3xTg-AD mice^45^ and from its WT counterpart. For immortalization, we used transduction with SV40 large T antigen, a protocol that has been extensively used previously^14,46,47^. During exploitation of the protocol we did not proceed with clonal selection, but instead continued growing and expanding a population of transduced cells. This allowed us to avoid the inter-clonal heterogeneity that is a characteristic of clonal selection^48–50^. Validity of this approach was efficiently demonstrated in gene expression and proteomics analyses, in which four independently generated iAstro lines for both WT and 3Tg-AD genotypes were analyzed and gave consistent results, and also demonstrated that 3Tg-iAstro retains the differences as compared with WT-iAstro found previously in primary cultures^40^.

To validate our model we used a number of methods including immunocytochemistry, real-time PCR (RT-PCR), electrophysiology and Ca^2+^ measurements. We show that the WT-iAstro cell lines express the astrocytic markers AQP4, GS and Aldh1l1, although at lower levels compared to primary cells and have electrophysiological properties comparable to primary astrocytes, at least as determined by evaluation of the Kir current. Similarly, real-time PCR of selected genes as well as mass spectrometry for proteins shows that the expression profile is similar to primary astrocytes. For a quantitative estimation of the astrocytic phenotype of iAstro lines, we have compared our proteomics data with similar data obtained on primary astroglial cultures. For this we used two lists reporting data from primary cultured rat^43^ and mouse^44^ cortical astrocytes. When our list was compared with the above-mentioned datasets, relatively high percentage of common proteins was found (65% of those reported by Hanrieder et al.^43^ and 74% of those reported by Yang et al.^44^), indicating that our list efficiently covers both analyzed datasets. This further confirms that after immortalization procedure, iAstro retains an astroglial phenotype. The only difference of note observed was a small proportion of GFAP-positive cells.

Having established that iAstro cell lines replicate primary culture, we next evaluated whether the changes observed in AD models could be replicated by the 3xTg-iAstro cells. For this, we capitalized on our previous data on Ca^2+^ signaling and on transcriptional profiling. Deregulations of astroglial Ca^2+^ signaling in AD have been reported^30,51–53^. Our group has demonstrated that primary hippocampal astrocytes from 3xTg-AD mice exhibit enhanced ATP-induced Ca^2+^ signals^34^ and SOCE^39^. In line with results on primary cultures, 3Tg-iAstro exhibited significantly higher amplitude of Ca^2+^ signals in response to ATP stimulation which is accompanied by enhanced SOCE. Analyzing components of the purinergic Ca^2+^ signaling we noticed that P2ry2 ATP-sensitive receptor is significantly up-regulated in both primary 3Tg astrocytes^40^ and in 3Tg-iAstro lines, corroborating the Ca^2+^ imaging data presented in Fig. 4. Notably, none of iAstro lines responded to DHPG or glutamate (not shown), suggesting that function or/and expression of mGluR5 may be hampered by the immortalization process.

We used the iAstro model for proteomics to demonstrate its usefulness and to add information on the role of astrocytes in AD. GO analysis of proteins down-regulated in 3Tg-iAstro as compared to WT-iAstro lines suggests that translation may be impaired in 3Tg-iAstro lines in two manners: (1) formation of nuclear ribonucleoprotein complex; and (2) formation of ribosomal multi-protein complex. Protein synthesis has already been suggested to be impaired in AD^54–56^ and recent findings suggest that it may occur early in AD pathogenesis^57^. In AD hippocampi, down-regulation of several proteins involved in chromatin compacting and regulation of rRNA transcription has been reported^58^ including nucleolin, which is also present in our list. Ribosomes are composed of the ribosomal RNAs and the ribosomal proteins that form the small subunit (40S) which binds to mRNA and the large subunit (60S) which binds to transfer RNAs and amino acids. Small subunit contains 33 proteins of which 3 (9%) are present in our list of down-regulated proteins, while large subunit contains 46 proteins of which 8 (17.3%) are present in our list, altogether suggesting that alterations in translation may represent an early astrocyte-specific event in AD pathogenesis^57^. Finally, we acknowledge that the straightforward translation of the results obtained on immortalized astrocytes in in vitro experiments to human AD pathogenesis is a rather speculative over-simplification. Further experiments, aiming at investigating the mechanistic aspects and confirmation of these results in vivo, are necessary to validate the presented data.

Transcriptome analysis of astrocytes freshly isolated from the brain of APPswe/PS1d9ex AD model mice at a symptomatic stage^59^, as well as of hippocampal cultured astrocytes from 3xTg-AD mice^40^, shows significant enrichment of genes in GO terms related to Ca^2+^. Proteomics data, however, do not reveal an overrepresentation of GO terms related to Ca^2+^. This may, at least in part, be explained by the fact that the shotgun mass spectrometry, used in this work, allows detection of only the most abundant proteins in iAstro lines. None of the proteins of the classical Ca^2+^ signaling toolkit, like Ca^2+^ transporters, channels or receptors, including P2Y2 purinergic receptor, has been detected. It is worth noting that the highest up-regulated protein in our analysis is calmodulin (CaM) (Table 1), whose role is to sense fluctuation of Ca^2+^ concentrations^60,61^. Downstream events include either transmission of the information to Ca^2+^-regulated signaling hubs, like Ca^2+^/CaM-activated kinases (CaMKs) of phosphatase calcineurin, or direct modulation of the activity of proteins, e.g., plasma membrane Ca^2+^ ATPase. Therefore, it is reasonable to suggest that 3.6-fold CaM overexpression in 3Tg-iAstro as compared to WT-iAstro (Table 1) may alter the entire Ca^2+^-dependent cellular homeostasis. While characterization of CaM-dependent processes in iAstro lines lies beyond the scope of present work, it is worth noting that in AD-related research, CaM-activated enzymes like CaMKII or calcineurin have been recurrently mentioned as central elements of disease pathogenesis and represent possible pharmacological targets^41,62,63^. Last, it has been recently suggested that CaM binds with high affinity to Aβ, thus representing a direct target for toxic Aβ peptide^64^. In light of our observations, our data warrant further detailed examination of the role of CaM in AD.

In conclusion, we have immortalized and characterized hippocampal astrocytes from WT and 3xTg-AD mouse pups. Using complementary methodologies we show that iAstro lines retain astroglial pattern of protein expression, retain fundamental astroglial housekeeping functions and show transcriptional and functional alterations found previously in primary astrocytes from 3xTg-AD mice compared to WT mice. In addition, proteomic analysis suggests that protein synthesis, due to impaired nuclear RNA binding and alterations in ribosome composition, may be specifically impaired in AD astrocytes. Altogether, our results suggest that 3Tg-iAstro may be a useful tool to study astrocyte-related alterations in AD.

## Materials and methods

### Animals

3xTg-AD mice used in this work were introduced by Frank LaFerla, Salvatore Oddo and colleagues in 2003^45^. These mice were developed on the mixed 129/C57BL6 background bearing knock-in mutation PS1_M146V_^65^ and in which APP_swe_ and Tau_P301L_ transgenes were introduced. The 3xTg-AD animals show major histological hallmarks of AD represented by senile plaques and neurofibrillary tangles. These mice show progressive learning and memory deficit beginning from 4 months of age^66^. The 3xTg-AD mice and their respective non-transgenic controls (WT)^45^ were housed in the animal facility of the Università del Piemonte Orientale, were kept at three to four per cage and had unlimited access to water and food. Animals were managed in accordance with the European directive 2010/63/UE and with Italian law D.l. 26/2014. The procedures were approved by the local animal-health and ethical committee (Università del Piemonte Orientale) and were authorized by the national authority (Istituto Superiore di Sanità; authorization number N. 22/2013). All efforts were made to reduce the number of animals by following the 3R (replacement, reduction and refinement) rule.

### Primary astroglial cultures preparation and astrocyte purification

For primary astroglial cultures, WT and 3xTg-AD P0-P2 pups were killed by decapitation. Hippocampi were rapidly dissected and minced with a scalpel blade in cold calcium- and magnesium-free Hank’s Balance Salt Solution (Sigma-Aldrich). The tissues were then digested with trypsin (Sigma-Aldrich; 0.25%, 37 °C) and triturated with 30 strokes of an automatic pipette. Non-dissociated tissue was allowed to sediment for 2 min and cell suspension was centrifuged for 5 min (200 × *g*), resuspended in complete culture medium (Dulbecco’s modified Eagle’s medium (DMEM; Sigma-Aldrich, Cat. No. D5671) supplemented with 10% fetal bovine serum (Gibco, Cat. No. 10270), 2 mM L-glutamine (Sigma-Aldrich), and 1% penicillin/streptomycin solution (Sigma-Aldrich) and plated in 60 mm Petri dishes (Falcon) (hippocampi from 3–6 pups per dish). Cells were maintained in a 5% CO_2_ 37 °C incubator. At ~90% of confluence, cells were detached and microglial cells were removed by MACS using anti-CD11b-conjugated beads (Miltenyi Biotech, Cat. No. 130-093-634). Purified astrocytes were collected, resuspended in complete culture medium and plated in a 35 mm dish for immortalization.

### Production of SV40-containing replication-defective retroviral vectors

Phoenix cells producing a replication-defective retrovirus^67^, grown in 100 mm culture dishes (Falcon, 1.5 × 10^6^ cells per dish), were transfected with pBABE-neo encoding neomycin phosphotransferase and SV40 large T antigen (Addgene, plasmid ID 1780^68^), using Lipofectamine 2000 reagent (Life Technologies, Segrate, Italy), according to the manufacturer’s instructions. At 48 h after transfection, cell medium containing the retroviral vectors was collected and filtered through 0.4 μm filters. The retroviral particles were precipitated by polyethylene glycol (O/N, 4 °C) prepared as described elsewhere^69^. The precipitate was concentrated by centrifugation (3500 × *g*, 30 min, 4 °C), the supernatant was discarded and the pellet was resuspended in complete culture medium (200 μl per 8 ml of initial medium), divided in 100 μl aliquots and stored at −80 °C.

### Astrocyte immortalization

MACS-purified hippocampal astrocytes were plated in 35 mm culture dishes (2.5 × 10^5^ cells per dish) and 24 h later were transduced with retrovirus expressing SV40 large T antigen. To increase the infection efficiency, dishes were shaken (80 rpm) overnight in a cell incubator. After 24 h, fresh medium was added for 72 h and then replaced with medium containing 0.4 mg/ml G418 disulfate salt solution (Sigma-Aldrich)^70^. At 3 to 4 weeks after cell selection, surviving cells were first sub-cultured in 100 mm dishes, expanded and maintained in complete culture medium supplemented with 0.4 mg/ml G418 until passage 10. After thawing from cryopreservation, iAstro lines were maintained in complete culture medium without G418 and used for experiments between passages 12 and 20.

### Immunofluorescence

WT- and 3Tg-iAstro cells, grown on 13 mm glass coverslips, were fixed in 4% formaldehyde, permeabilized (7 min in 0.1% Triton X-100 in phosphate-buffered saline (PBS)) and immunoprobed with an appropriate primary antibody (diluted in PBS supplemented with 1% gelatin) for 1 h at 37 °C. After 3 times washing in PBS, an Alexa-conjugated secondary antibody (1:300 in PBS supplemented with 1% gelatin) was applied for 1 h at room temperature (RT). The following primary antibodies were used: AQP4 (Alomone Labs, Cat. No. 249-323), Aldh1l1 (Abcam, Cat. No. Ab190298), GS (Abcam, Cat. No. Ab73593), GFAP (Chemicon International, Cat. No. CBL411) and GLT-1 (Alomone labs, Cat. No. AGC-022). Secondary antibodies were as follows: Alexa Fluor 488 anti-mouse IgG, Alexa Fluor 555 anti-rabbit IgG (all secondary antibodies were from Molecular Probes, Life Technologies, Monza, Italy). Nuclei were counter-stained with 4′,6-diamidino-2-phenylindole (DAPI). Images were acquired by Zeiss 710 confocal laser scanning microscope equipped with EC Plan-Neofluar 40×/1.30 Oil DIC M27 objective and Zen software.

### Total RNA extraction and real-time PCR

Total RNA was extracted from 1 × 10^6^ cells using Absolutely RNA miRNA kit (Agilent, Santa Clara, CA) according to the manufacturer’s instructions. Total RNA (0.5–1 μg) was retro-transcribed using random hexamers and ImProm-II RT system (Promega, Milan, Italy). Real-time PCR was performed using iTaq qPCR master mix according to the manufacturer’s instructions (Bio-Rad, Segrate, Italy) on a SFX96 Real-time system (Bio-Rad). To normalize raw real-time PCR data, S18 ribosomal subunit was used. Sequences of oligonucleotide primers are provided in Supplementary Materials. The real-time PCR data are expressed as delta-C(t) of gene of interest to S18 allowing appreciation of the relative expression level of a single gene.

### Electrophysiological recordings

To perform patch-clamp experiments in whole-cell configuration, both WT-iAstro and 3Tg-iAstro were plated separately in 35 mm dishes 24 h prior experiments. Cells were plated at low density to allow recordings from isolated astrocytes. They were transferred from culture medium to an extracellular solution containing (in mM): 138 NaCl, 4 KCl, 2 CaCl_2_, 1 MgCl_2_, 10 glucose and 10 HEPES at pH 7.25 adjusted with NaOH. Borosilicate patch pipettes were pulled with a P-1000 puller (Sutter Instruments, USA) and were filled with a solution containing (in mM): 140 KCl, 2 NaCl, 5 EGTA, 0.5 CaCl_2_ and 10 HEPES at pH 7.25 adjusted with KOH. Pipette tip resistance containing this solution was between 3 and 5 MΩ. Experiments were performed using an EPC7 Plus amplifier (HEKA Elektronik, Germany) in voltage-clamp configuration (holding potential, –80 mV). Access resistance (8–12 MΩ) was compensated (80–90%) and experiments were performed at RT and in a static bath. Data were acquired at 5 kHz and filtered at 1 kHz using a 7-pole Bessel filter and digitized with a low noise data acquisition system, Digidata 1440A (Molecular Devices, USA). Data were recorded and analyzed in pClamp 10 (Molecular Devices, Crisel Instruments, Italy). Data were initially processed with Microsoft Excel. Plots, bar diagrams and figure preparations were finalized with GraphPad Prism (GraphPad Software, La Jolla, CA).

### Cell lysates

WT- and 3Tg-iAstro cells were plated at a density of 2 × 10^5^ cells/100 mm dish and cultured until 80–90% of confluence. Then, cells were washed twice with cold PBS and lysed in 800 µl lysis buffer (50 mM Tris-HCl pH 7.4, 150 mM NaCl, 0,5 mM EDTA, 0,1% NP40, 0,1% SDS) supplemented with protease and phosphatase inhibitors cocktail (Thermo Scientific Halt Protease and Phosphatase Inhibitor Cocktail, Cat. No. 78444). For each sample, protein concentration was measured by BCA assay (Quanti Pro BCA Asssay Kit, SIGMA, Cat. No. QPBCA-1Kt) and read by Bio-Rad SMART^TM^Plus Spectrophotometer.

### Western blotting

Specific astroglial proteins were detected in WT- and 3Tg-iAstro lysates by western blotting (WB). Each sample, containing 100 µg of protein, was diluted 1:1 in 2× Laemmli sample buffer, heated at 95 °C for 5 min, then resolved by sodium dodecyl sulfate–polyacrylamide gel electrophoresis (SDS-PAGE). Proteins were electrophoretically transferred to nitrocellulose membranes and the membranes were blocked for 1 h in 5% (w/v) non-fat dry milk in Tris-buffered saline containing Tween-20. After incubation with appropriate primary and secondary antibodies, signals were revealed using enhanced chemiluminescence (Super Signal West Femto Maximum Sensitivity Substrate, Thermo Scientific, Cat. No. 34095), and were visualized by a Bio-Rad ChemiDoc touch Imaging system. Anti-AQP4 (1:1000, Alomone Labs, Cat. 249-323), anti-GS (1:2000, Abcam, Cat. No. Ab73593), anti-Aldh1l1 (1:2000, Abcam, Cat. No. Ab190298) and anti-β-actin (1:10,000, Sigma, Cat. No. A1978) antibodies were used to develop western blots, as indicated in the figure legends. Densitometric analysis was performed using Quantity One software and is expressed as mean ± SEM from at least 3 independent runs. Analysis of variance (ANOVA) test was used for statistical analysis. Note relatively big variability between technical replicates in WB analysis, which may be explained by low level of specific astroglial proteins expressed in iAstro lines.

### Fura-2 calcium imaging

For Ca^2+^ imaging, iAstro lines grown onto 24 mm round coverslips were loaded with Fura-2/AM (Life Technologies, Milan, Italy, Cat. No. F1201) in the presence of 0.005% Pluronic F-127 (Life Technologies, Cat. No. P6867) and 10 μM sulfinpyrazone (Sigma, Cat. No. S9509) in KRB solution (125 mM NaCl, 5 mM KCl, 1 mM Na_3_PO_4_, 1 mM MgSO_4_, 5.5 mM glucose, 20 mM HEPES, pH 7.4) supplemented with 2 mM CaCl_2_. After loading and 30 min of de-esterification, the coverslips were mounted in an acquisition chamber on the stage of a Leica epifluorescence microscope equipped with a S Fluor 40×/1.3 objective. Cells were alternatively excited at 340/380 nm by the monochromator Polichrome V (Till Photonics, Munich, Germany) and the fluorescent signal was collected by a CCD camera (Hamamatsu, Japan) through bandpass 510 nm filter; the experiments were controlled and images analyzed with MetaFluor (Molecular Devices, Sunnyvale, CA, USA) software. The cells were stimulated by 20 μM ATP. To quantify the difference in the amplitude of Ca^2+^ transients, the ratio values were normalized according to the formula (ΔF)/F_0_ (referred to as norm. Fura ratio).

### Proteomic analysis

#### In solution digestion

Cell lysates were digested using the following protocol: samples were prepared to have 100 μg of protein in a final volume of 25 μl of 100 mM NH_4_HCO_3_. Proteins were reduced using 2.5 μl of dithiothreitol (200 mM DTT stock solution) (Sigma) at 90 °C for 20 min, and alkylated with 10 μl of Cysteine Blocking Reagent (iodoacetamide (IAM), 200 mM Sigma) for 1 h at room temperature in the dark. DTT stock solution was then added to destroy the excess of IAM. After dilution with 300 μl of water and 100 μl of NH_4_HCO_3_ to raise pH 7.5–8.0, 5 μg of trypsin (Promega, Sequence Grade) was added and digestion was performed overnight at 37 °C. Trypsin activity was stopped by adding 2 μl of neat formic acid and samples were dried by Speed Vacuum^71^.

The peptide digests were desalted on the Discovery® DSC-18 solid-phase extraction (SPE) 96-well Plate (25 mg/well) (Sigma-Aldrich Inc., St. Louis, MO, USA). The SPE plate was preconditioned with 1 ml of acetonitrile and 2 ml of water. After the sample loading, the SPE was washed with 1 ml of water. The adsorbed proteins were eluted with 800 μl of acetonitrile/water (80:20). After desalting, samples were vacuum evaporated and reconstituted with 20 μl of 0.05% formic acid in water. Then, 2 μl of stable-isotope-labeled peptide standard (DPEVRPTSAVAA, Val- 13C5 15N1 at V10, Cellmano Biotech Limited, Anhui, China) was spiked into the samples before the liquid chromatography–tandem mass spectrometry (LC-MS/MS) analysis and used for instrument quality control.

#### Label-free proteomic analysis

LC-MS/MS analyses were performed using a micro-LC Eksigent Technologies (Dublin, USA) system with a stationary phase of a Halo Fused C18 column (0.5 × 100 mm, 2.7 μm; Eksigent Technologies, Dublin, USA). The injection volume was 4.0 μl and the oven temperature was set at 40 °C. The mobile phase was a mixture of 0.1% (v/v) formic acid in water (A) and 0.1% (v/v) formic acid in acetonitrile (B), eluting at a flow rate of 15.0 μl/min at an increasing concentration of solvent B from 2 to 40% in 30 min. The LC system was interfaced with a 5600+TripleTOF system (AB Sciex, Concord, Canada) equipped with a DuoSpray Ion Source and CDS (Calibrant Delivery System). Samples used to generate the SWATH-MS (sequential window acquisition of all theoretical mass spectra) spectral library were subjected to the traditional data-dependent acquisition (DDA): the mass spectrometer analysis was performed using a mass range of 100–1500 Da (TOF scan with an accumulation time of 0.25 s), followed by a MS/MS product ion scan from 200 to 1250 Da (accumulation time of 5.0 ms) with the abundance threshold set at 30 cps (35 candidate ions can be monitored during every cycle). Samples were then subjected to cyclic data-independent analysis (DIA) of the mass spectra, using a 25 Da window. A 50 ms survey scan (TOF-MS) was performed, followed by MS/MS experiments on all precursors. These MS/MS experiments were performed in a cyclic manner using an accumulation time of 40 ms per 25 Da swath (36 swaths in total) for a total cycle time of 1.5408 s. The ions were fragmented for each MS/MS experiment in the collision cell using the rolling collision energy. The MS data were acquired with Analyst TF 1.7 (SCIEX, Concord, Canada). Three instrumental replicates for each sample were subjected to the DIA analysis^72,73^.

#### Protein database search

The mass spectrometry files were searched using Protein Pilot (AB SCIEX, Concord, Canada) and Mascot (Matrix Science Inc., Boston, USA). Samples were input in the Protein Pilot software v. 4.2 (AB SCIEX, Concord, Canada), which employs the Paragon algorithm, with the following parameters: cysteine alkylation, digestion by trypsin, no special factors and false discovery rate (FDR) at 1%. The UniProt Swiss-Prot reviewed database containing mouse proteins (version 20july15, containing 23,304 sequence entries). The Mascot search was performed on Mascot v. 2.4, the digestion enzyme selected was trypsin, with 2 missed cleavages and a search tolerance of 50 ppm was specified for the peptide mass tolerance, and 0.1 Da for the MS/MS tolerance. The charges of the peptides to search for were set to 2+, 3+ and 4+, and the search was set on monoisotopic mass. The instrument was set to ESI-QUAD-TOF (electrospray ionization quadrupole time-of-flight) and the following modifications were specified for the search: carbamidomethyl cysteines as fixed modification and oxidized methionine as variable modification.

#### Protein quantification

The quantification was performed by integrating the extracted ion chromatogram of all the unique ions for a given peptide. The quantification was carried out with PeakView 2.0 and MarkerView 1.2. (Sciex, Concord, ON, Canada). Six peptides per protein and six transitions per peptide were extracted from the SWATH files. Shared peptides were excluded as well as peptides with modifications. Peptides with FDR lower than 1.0% were exported in MarkerView for the *t*-test.

#### Analysis of identified proteins

For identification of the number of detected proteins for each genotype, the intersection of four lists, corresponding to four analyzed lines per genotype, was performed, yielding lists of common proteins detected in four WT- and four 3Tg-iAstro lines (1119 and 1045, respectively). The intersection of the latter two lists gave proteins detected in both WT- and 3Tg-iAstro cells (856 proteins).

### Gene ontology analysis

GO analysis was performed using DAVID v.6.8 tool (https://david.ncifcrf.gov/)^74^. For the analysis of the overrepresented GO terms, for the background, a list containing all proteins detected in WT-iAstro and 3Tg-iAstro lines (1308 proteins) was used. Over-represented GO terms which passed Benjamini correction (*p* < 0.05) were considered significant. For prediction of protein–protein interactions and clustering using *k*-means algorithm, STRING v.10.5 online software was used (https://string-db.org/)^75^.

### Glutamate uptake

Primary astrocytes, WT and 3Tg-iAstro lines were cultured in 24-well plates at 5 × 10^4^ cells /well. Cells were treated in DMEM/F12 medium with 5 mM glutamate (l-glutammic acid momosodium salt, Sigma, Cat. No. G1626) with or without TBOA ((3S)-3-[[3-[[4-(trifluoromethyl)bemzoyl]amino]phenyl]methoxy]-l-aspartic acid, Tocris, Cat. No. 2532). After 2 h, supernatants were collected, centrifuged (16,000 × *g* for 10 min at 4 °C) and analyzed for residual glutamate using the protocol described by the manufacturers (Amplex® Red Glutamic Acid/Glutamate Oxidase Kit, Invitrogen, Cat. No. A12221). Glutamate uptake was calculated as difference of fluorescence at 590 nm between glutamate plus TBOA and glutamate alone and normalized to protein concentrations of corresponding cell lysate.

### LPS and TNFα treatment

For treatment with bacterial LPS (lipopolysaccharides from *Escherichia coli* O111:B4, Sigma, Cat. No. L2630) and TNFα (Peprotech, London, UK, Cat. No. 300-01A), cells were plated in 12-well plates (1 × 10^5^ cells per well) and, upon confluence, were treated with either LPS (for 3 h) or TNFα (for 6 h). The cells were then lysed in 500 μl Trizol Reagent (Life Technologies) and total RNA was extracted according to the manufacturer’s instructions. First-strand complementary DNA and real-time PCR were performed as described above. Oligonucleotide primers for iNOS are listed in Supplementary Materials.

### Experimental groups selection and statistical analysis

For the selection of experimental groups, the following criteria were adopted: in experiments in which entire populations of cells were analyzed (western blotting, real-time PCR and proteomic analysis), experimental group was composed of 4 independently generated iAstro lines for each genotype (WT-iAstro#2, #3, #5 and #6, and 3Tg-iAstro#2, #3, #4 and #6) representing biological replicates. Technical replicates consisted of at least three independent experiments of each iAstro line. In experiments, in which single-cell analysis was performed (immunocytochemistry, electrophysiology and Fura-2 Ca^2+^ imaging), experimental groups were composed of independent experiments (at least three coverslip (technical replicates) from at least three different experiments) of one line per genotype were used (WT-iAstro#2 and 3Tg-iAstro#2).

Statistical analysis was performed using GraphPad Prism software v.7. For analysis of real-time PCR and Ca^2+^ imaging data, a two-tailed unpaired Students’s *t-*test was used. For western blot ANOVA on raw followed by Tukey’s post-hoc test was used. Differences were considered significant at *p* < 0.05.

## Supplementary information

Supplementary material

Supplementary Table 1

Supplementary Table 2A

Supplementary Table 2B

Supplementary Table 3

Supplementary Table 4

Supplementary Table 5
