# Correction: Gene expression, proteome and calcium signaling alterations in immortalized hippocampal astrocytes from an Alzheimer’s disease mouse model

**DOI:** 10.1038/s41419-020-2451-y

**Published:** 2020-04-16

**Authors:** Francesca Rocchio, Laura Tapella, Marcello Manfredi, Mariangela Chisari, Francesca Ronco, Federico Alessandro Ruffinatti, Eleonora Conte, Pier Luigi Canonico, Maria Angela Sortino, Mariagrazia Grilli, Emilio Marengo, Armando A. Genazzani, Dmitry Lim

**Affiliations:** 1 Department of Pharmaceutical Sciences, Università degli Studi del Piemonte Orientale, Novara, Italy; 2 Department of Sciences and Technological Innovation, Università degli Studi del Piemonte Orientale, Alessandria, Italy; 3 ISALIT S.r.l., Spin-off of Università degli Studi del Piemonte Orientale, Novara, Italy; 4 Department of Biomedical and Biotechnological Sciences, Section of Pharmacology, University of Catania, Via Santa Sofia, 97, 95123 Catania, Italy; 5 Present Address: International Center for T1D, Pediatric Clinic Research Center Fondazione Romeo ed Enrica Invernizzi, Department of Biomedical and Clinical Science L. Sacco, University of Milan, Milan, Italy

**Keywords:** Astrocyte, Alzheimer's disease

Correction to: *Cell Death and Disease*

10.1038/s41419-018-1264-8

published online 10 January 2019

In the original published version of this article, Fig. 1a included an incorrect image. The correct image is as follows:

**Figure 1:**
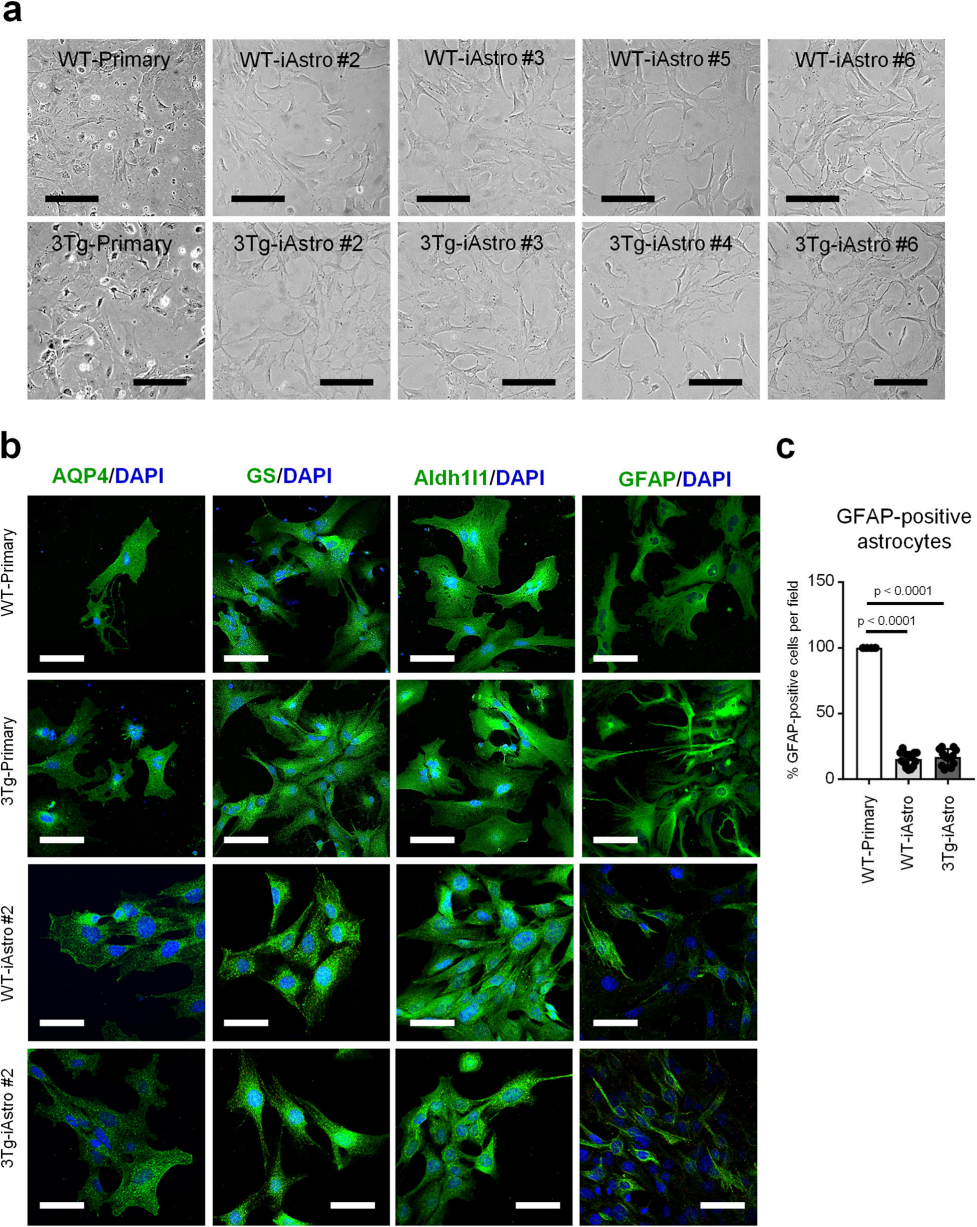
Fig. 1

This has been corrected in both the PDF and HTML versions.

