# Supplementary material for: Gene expression, proteome and calcium signaling alterations in immortalized hippocampal astrocytes from an Alzheimer’s disease mouse model
Source: Cell Death Dis. 2019 Jan 10;10(1):24. doi: 10.1038/s41419-018-1264-8 (PMC6328590; doi:10.1038/s41419-018-1264-8)
Supplement: Supplementary file 1 — Supplementary material [file 41419_2018_1264_MOESM1_ESM.pdf]

SUPPLEMENTARY MATERIAL

Gene expression, proteome and calcium signaling alterations in immortalized hippocampal astrocytes from an Alzheimer's disease mouse model.

Francesca Rocchio, Laura Tapella, Marcello Manfredi, Mariangela Chisari, Francesca Ronco, Federico Alessandro Ruffinatti, Eleonora Conte, Pier Luigi Canonico, Maria Angela Sortino, Mariagrazia Grilli, Emilio Marengo, Armando A Genazzani and Dmitry Lim

Index

File or Table	Content
Supplementary Material	Oligonucleotide primers used for real-time PCR
Supplementary Figure 1	GLT-1 expression and glutamate uptake
Supplementary Figure 2	LPS-induced iNOS and TNFα mRNA levels
Supplementary Figure 3	TGFβ mRNA levels in WT-iAstro and 3Tg-iAstro lines
Supplementary Table 1	List of detected proteins in WT- and 3Tg-iAstro lines
Supplementary Table 2	Comparison of our dataset with datasets of Hanrieder et al., 2011 and Yang et al., 2005
Supplementary Table 3	List of differentially expressed proteins between WT- and 3Tg-iAstro immortalized astrocytes
Supplementary Table 4	Gene ontology analysis
Supplementary Table 5	String k-means clustering analysis

Oligonucleotide primers used for quantitative real-time PCR.

Gene	Accession number	Forward Reverse	Sequence 5' to 3'
Trim12a	NM_023835.2	Forward Reverse	TCATTGAAGAGGTGGCCCAG ACCGAACATTCTGCACATCT
Ccl27a (Pesky)	NM_001048179.1	Forward Reverse	TCTCCAACAAGCCAGAGACT GCTTGGGAGTGGCTGTCTAT
Car9	NM_139305.2	Forward Reverse	GGAGTCCCTTGGGTTAGAGG TGGGGCCAGAGTAGGGTG
Ptchd2	NM_001083342.1	Forward Reverse	CACAGCCTGCAGAACAATGT TTGGAGATGTACACGGTGCT
Slc2a4	NM_009204.2	Forward Reverse	TAAAACAAGATGCCGTCGGG CCAAACTGAAGGGAGCCAAG
P2ry2	NM_008773.3	Forward Reverse	ATCCTCACCACCTCAAGAGC GGCAACAGCACGTACTTGAA
Panx1	NM_019482.2	Forward Reverse	TCACATGTATTGCCGTGGGT CTCGGGGAGAAGCAGCTTAT
Cacna2d3	NM_009785.1	Forward Reverse	TGCCTGTGAACATCAGTCTGA GCCATATGAGAGACGGATCCC
Fbln2	NM_007992.2	Forward Reverse	GGGCACTCACGATTGTAGCT GCAGGTCTGTCACACACTCA
Col2a1	NM_001113515.2	Forward Reverse	GTGAGCCATGATCCGCCTC GGTTCTCCTTTCTGCCCCTT
Padi2	NM_008812.2	Forward Reverse	GGGAAAATATGCTGCGGGAAC CACCTTCCGAGTGCTTCAGG
Pcdh17	NM_001013753.2	Forward Reverse	CTAATTCGAGCGAGACCCCT CTCTCAGGCTGGCTCTTTCT
Samd4	NM_001037221.2	Forward Reverse	TTGTGCCTCAGTATGACGGA AACCCTGAAGATGGCTGACA
Col4a6	NM_053185.2	Forward Reverse	CAAGCATGCACCCTGGATTG AGACTCCATGGCAATCTCGG
P2ry1	NM_008772	Forward Reverse	TCAAGCAGAATGGAGACACG CTCACTCAGGTGGCACACAC
iNOS	NM_010927.4	Forward Reverse	GCCACCAACAATGGCAACA CGTACCGGATGAGCTGTGAATT
Tgfb2	NM_009367.3	Forward Reverse	AGGCAGAGTTCAGGGTCTTC CCTTGCTATCGATGTAGCGC
Tgfb3	NM_009368.3	Forward Reverse	AGGAGACCTCGGAGTCTGAG CACTGAGGACACATTGAAACGA
S18	NM_213557	Forward Reverse	TGCGAGTACTCAACACCAACA CTGCTTTCCTCAACACCACA

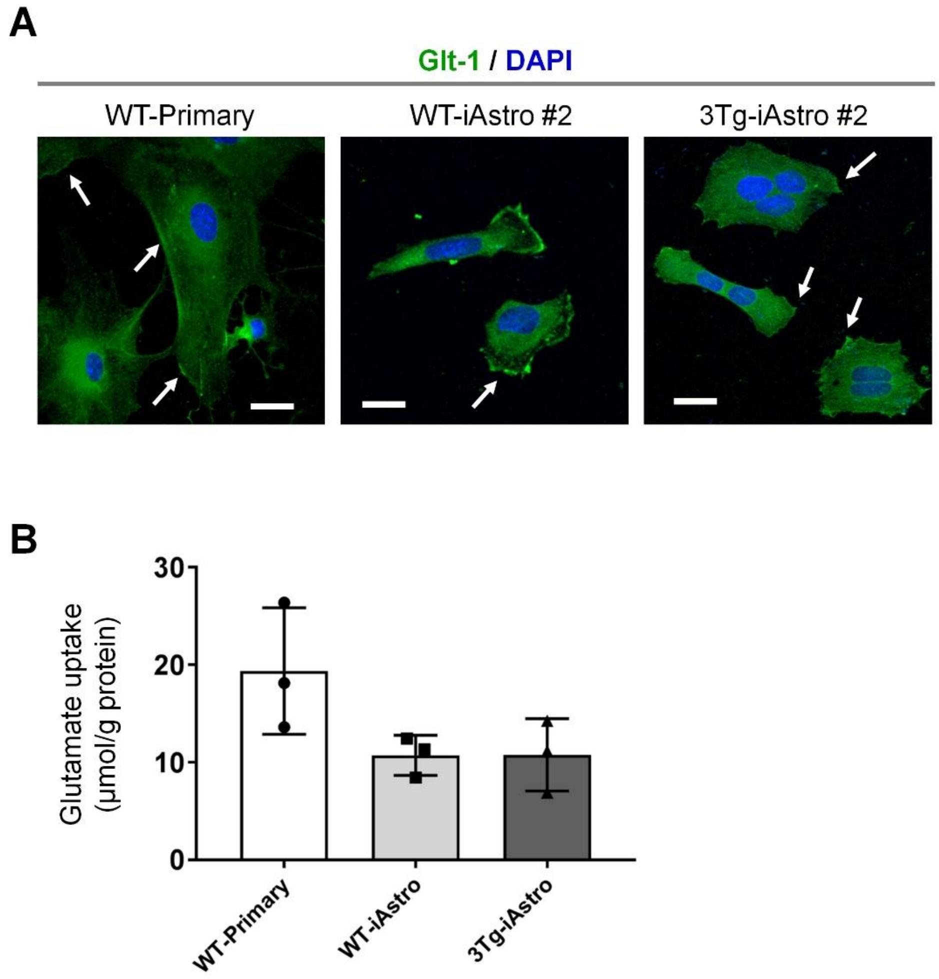

Supplementary Figure 1. iAstro express membrane-localized GLT-1 and are capable of glutamate uptake. A, membrane localization of GLT-1 in primary WT astrocytes and in WT-iAstro#2 and 3Tg-iAstro#2. The images are representative of 3 independent experiments. Similar staining was observed in other independently generated iAstro lines. Bar, 20 µm. **B**, Glutamate uptake was measured as described in Methods section in WT-primary and WT- and 3Tg-iAstro cells. The data are expressed as mean ± SD of experiments performed in triplicate for 3 independent cultures of WT primary astrocytes and WT-iAstro#2 and 3Tg-iAstro#2. ANOVA, followed by Tukey's post-hoc test was used for statistical analysis. The differences were not significant at $p < 0.05$.

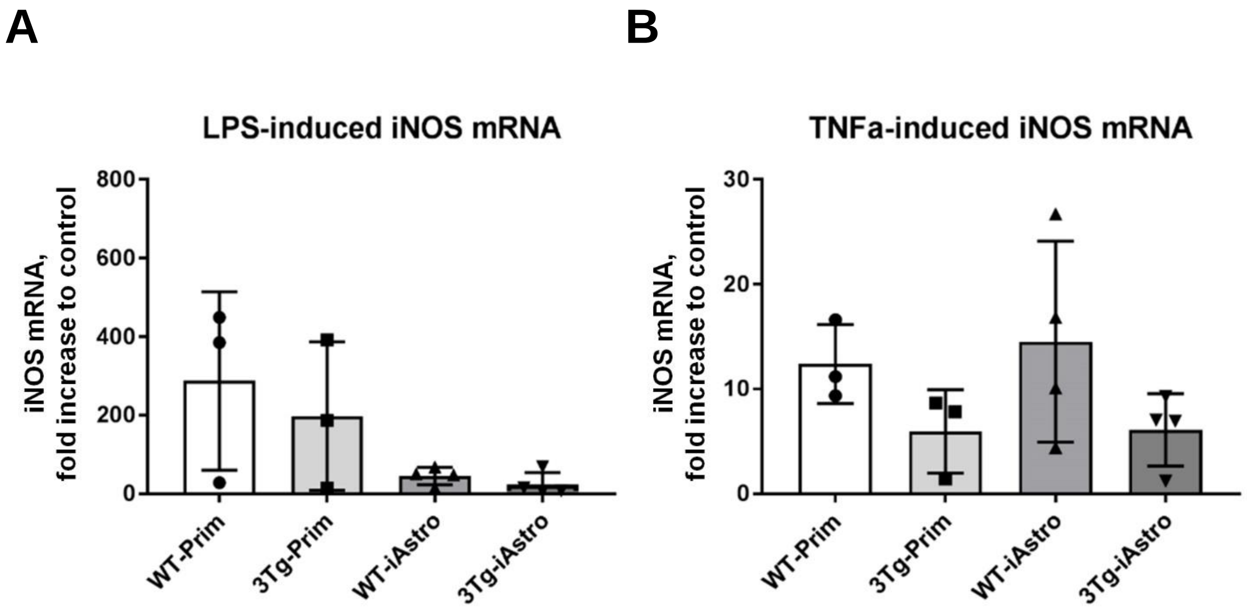

Supplementary Figure 2. LPS and TNFα-induced iNOS mRNA in WT- and 3Tg primary and immortalized astrocytes. Induction of iNOS upon treatment of primary and immortalized astrocytes from WT and 3xTg-AD mice with LPS (**A**) (100 ng/ml for 3h) or TNFα (**B**) (20 ng/ml 6h). Data are expressed as mean ± SD of iNOS mRNA fold increase as compared to WT cells of 3 independent cultures for primary astrocytes and 4 independently generated iAstro lines (WT-iAstro#2,#3,#5 and #6, and 3Tg-iAstro#2,#3,#4 and #6). ANOVA, followed by Tukey's post-hoc test was used for statistical analysis. The differences were not significant at $p < 0.05$.

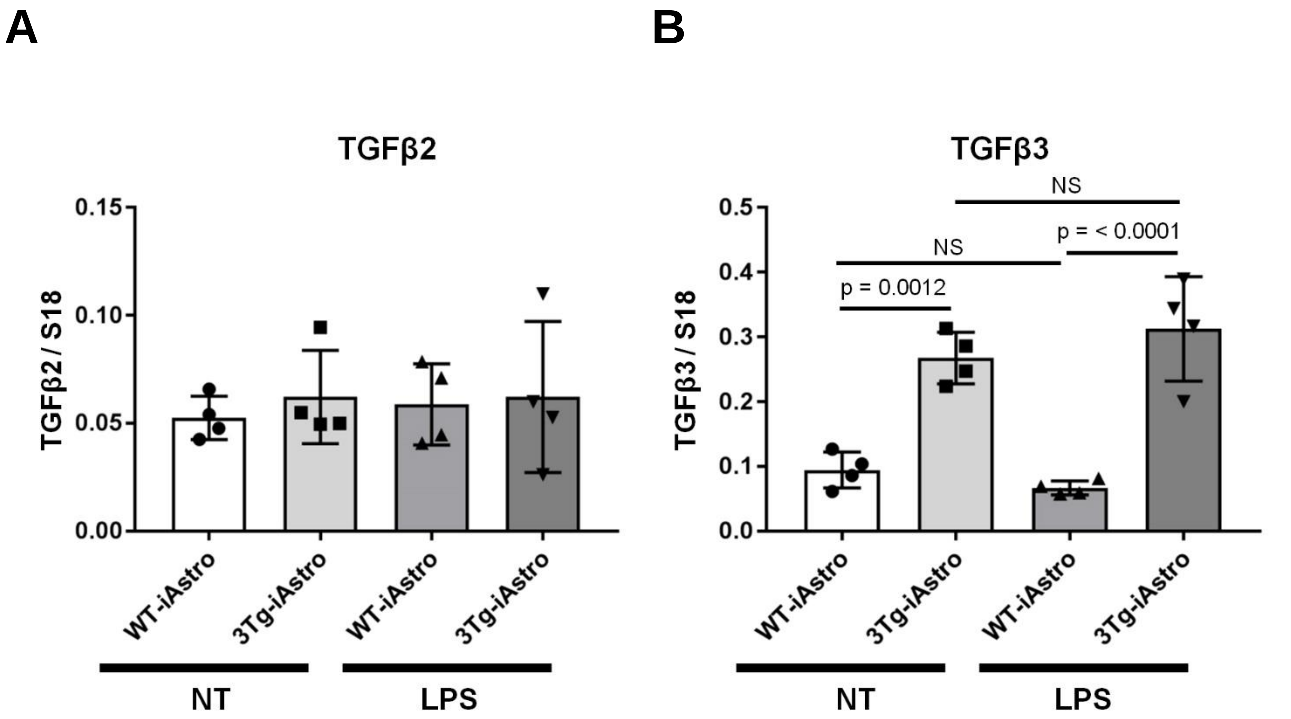

Supplementary Figure 3. TGFβ2 and TGFβ3 mRNA levels in WT-iAstro and 3Tg-iAstro. TGFβ2 (**A**) and TGFβ3 (**B**) mRNA levels were measured by real-time PCR in control non treated (NT) and LPS-treated (100 ng/ml, 3h) WT-iAstro and 3Tg-iAstro lines. Data are expressed as mean ± SD of ΔC(t) TGFβ/S18 mRNA of 4 independently generated iAstro lines (WT-iAstro#2,#3,#5 and #6, and 3Tg-iAstro#2,#3,#4 and #6). ANOVA, followed by Tukey's post-hoc test was used for statistical analysis. Where not indicated, the differences were not significant at $p < 0.05$.
